# Head and neck cancer predictive risk estimator to determine control and therapeutic outcomes of radiotherapy (HNC-PREDICTOR): development, international multi-institutional validation, and web implementation of clinic-ready model-based risk stratification for head and neck cancer

**DOI:** 10.1016/j.ejca.2022.10.011

**Published:** 2022-10-21

**Authors:** Lisanne V. van Dijk, Abdallah SR. Mohamed, Sara Ahmed, Nafiul Nipu, G. Elisabeta Marai, Kareem Wahid, Nanna M. Sijtsema, Brandon Gunn, Adam S. Garden, Amy Moreno, Andrew J. Hope, Johannes A. Langendijk, Clifton D. Fuller

**Affiliations:** a Department of Radiation Oncology, The University of Texas MD Anderson Cancer Center, Houston, TX, USA; b Department of Radiation Oncology, University Medical Center Groningen, University of Groningen, Groningen, the Netherlands; c Department of Computer Science, The University of Illinois Chicago, Chicago, USA; d MD Anderson Stiefel Center for Oropharyngeal Cancer Research and Education (MDA-SCORE), Houston, TX, USA; e Department of Radiation Oncology, University of Toronto, Toronto, Canada; f Radiation Medicine Program, Princess Margaret Cancer Centre, University Health Network, Toronto, Canada

**Keywords:** Head and neck cancer, Overall survival, Machine learning, Image biomarkers, Decision support tool

## Abstract

**Background:**

Personalised radiotherapy can improve treatment outcomes of patients with head and neck cancer (HNC), where currently a ‘one-dose-fits-all’ approach is the standard. The aim was to establish individualised outcome prediction based on multi-institutional international ‘big-data’ to facilitate risk-based stratification of patients with HNC.

**Methods:**

The data of 4611 HNC radiotherapy patients from three academic cancer centres were split into four cohorts: a training (n = 2241), independent test (n = 786), and external validation cohorts 1 (n = 1087) and 2 (n = 497). Tumour- and patient-related clinical variables were considered in a machine learning pipeline to predict overall survival (primary end-point) and local and regional tumour control (secondary end-points); serially, imaging features were considered for optional model improvement. Finally, patients were stratified into high-, intermediate-, and low-risk groups.

**Results:**

*Performance score, AJCC*^*8th*^ *stage, pack-years,* and *Age* were identified as predictors for overall survival, demonstrating good performance in both the training cohort (c-index = 0.72 [95% CI, 0.66–0.77]) and in all three validation cohorts (c-indices: 0.76 [0.69–0.83], 0.73 [0.68–0.77], and 0.75 [0.68–0.80]). Excellent stratification of patients with HNC into high, intermediate, and low mortality risk was achieved; with 5-year overall survival rates of 17–46% for the high-risk group compared to 92–98% for the low-risk group. The addition of morphological image feature further improved the performance (c-index = 0.73 [0.64–0.81]). These models are integrated in a clinic-ready interactive web interface: https://uic-evl.github.io/hnc-predictor/

**Conclusions:**

Robust model-based prediction was able to stratify patients with HNC in distinct high, intermediate, and low mortality risk groups. This can effectively be capitalised for personalised radiotherapy, e.g., for tumour radiation dose escalation/de-escalation.

## 1. Introduction

Head and neck cancer (HNC) affects almost 650,000 individuals and causes 350,000 deaths worldwide annually [1]. Historically, the main etiological HNC risk factor was smoking; hence, HNC incidence rates were expected to decrease along with the decline in societal smoking [2–5]. Yet, HNC cases increased due to a relatively new epidemiological subtype, human papilloma virus (HPV)-related HNC, which affects relatively younger patients and is associated with much better prognosis compared to HPV-negative HNC [6,7].

Radiotherapy is a cornerstone for curative HNC treatment. To date, a ‘one-dose-fits-all’ approach is deployed, i.e., all patients receive roughly similar tumour radiation dose prescription based mainly on historic pre-HPV clinical trials. Currently, personalising radiation dose to optimise tumour control is relatively unexplored. For instance, only tumour stage (i.e., early stage versus locally advanced) is used to select eligible patients in recent dose-escalation clinical trials, aiming to improve treatment control by increasing the radiation tumour dose [8–11]. The risk of severe radiation-induced sequelae from dose-escalation [10] makes improved selection a vital unmet need. On the other hand, patients with a low risk of treatment failure might benefit from de-intensified treatment, e.g., MR-guided dose de-escalation [12]. To date, attempts at therapeutic de-intensification in large heterogenous cohorts without patient-specific criteria have been uncompelling [13–15]; consequently, granular treatment outcome estimation for directed dose modification remains a substantive opportunity for HNC treatment personalisation.

Robust treatment outcome prediction based on multifactorial clinical variables is thus crucial to improve treatment success and establish effective personalised radiotherapy [16,17]. While clinical models have been developed [18–21], they are largely unused; clinical implementation has been hampered due to the lack of clinically useful prediction tools that are backed by large representative multi-institutional dataset for training and validation. Additionally, radiomics features – tumour-specific characteristics quantified from medical images – have been shown to improve HNC treatment outcome prediction [22–24]. An approach to add imaging features to well-established clinical models is needed for robust radiomics applications.

**Fig. 1.**
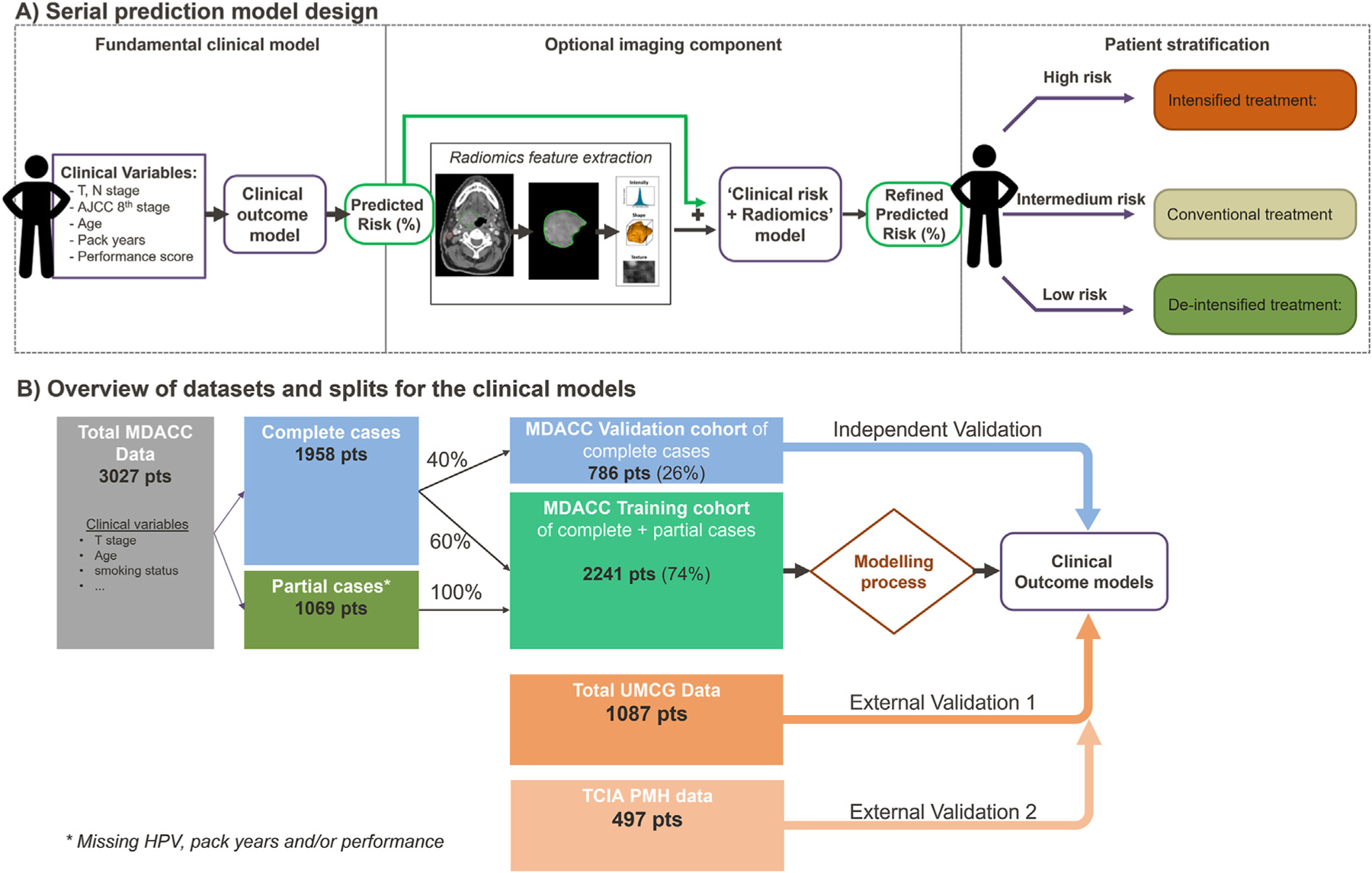
Study overview. *(A) Serial prediction model design.* The ‘fundamental clinical model’ component is the core component as it is based data of >4500 patients; the ‘predicted risk(%)’ can be refined with the ‘optional imaging component’, using radiomics features to improve the outcome risk prediction (‘refined Predicted Risk (%)’) to stratify patients in low-, intermediate-, and high-risk patients. The imaging component can be dynamically updated with future technical developments. (B) *Datasets for clinical model training, validation, and external validation.* Partial cases are patient that are missing at least one variable. Only complete cases were used for the validation of the models.

The main aim was to establish a large-scale multi-institutional standard for a more individualised outcome prediction in patients with HNC of overall survival (OS) and oncologic outcomes (i.e., local [LC] and regional control [RC]) following radiotherapy using large high-quality international datasets (>4500 patients with HNC). Additionally, an interactive web-based risk prediction tool was pursued to make the models direct clinically actionable for clinicians. Finally, we present a *serial* prediction model approach, where the clinical models can be enriched by *an optional imaging component* (Fig. 1A).

## 2. Methods

### 2.1. Patient considerations

The MD Anderson Cancer Center (MDACC) Big Data Radiotherapy HNC collection effort has been initiated for this study. The prospective and retrospective data collection was approved by the MDACC Institutional Review Board [PA14-0947/RCR03-0800]. This dataset was used for training and independent validation. Prospectively collected data from the University Medical Center Groningen (UMCG) were used for external validation (Standardized Follow-up Program: NCT02435576). The publicly available data from Princes Margret Hospital (PMH) on The Cancer Imaging Archive (TCIA) were used for additional external validation [25].

Inclusion criteria for all cohorts included: (1) proven squamous cell carcinoma of the head and neck, (2) treatment with definitive or adjuvant radiotherapy with/without chemotherapy, and (3) no prior head and neck radiation. Patients were treated from 2001 to 2019, 2007 to 2020, and 2005 to 2010 at MDACC, UMCG, and PMH, respectively. Prescribed tumour doses were 60–72 Gy, as detailed previously by each institution [23,26,27].

### 2.2. Outcome measures

The primary prediction end-point was OS. The secondary end-points were LC and RC, which were defined as recurrent, progressive, or residual disease of the primary tumour or regional lymph nodes after radiotherapy, respectively (with death as a censor). Time-to-event was measured from start of radiotherapy until the event, alternatively data were censored at last follow-up date. Systematic follow-up was part of the standard of care in both treatment centres: every 3 months in year 1, followed by every 6 months thereafter.

### 2.3. Clinical variables definitions

The clinical variables (and categorisations) considered in this study were demarcated as follows: gender (female, male); age (<55, 55–65, 65–75, >75); performance score (0, 1, ≥2); smoking status (current, former, never); pack-years (<5, 5–25, 26–50, >50); T-stage (T0-1, T2, T3, T4); N-stage (N0-2a/b, N2c, N3); tumour site (oropharynx [OPC], larynx, hypopharynx, nasopharynx, oral cavity); HPV status (positive and negative), and tumour stage *AJCC*^*8th*^ (I, II, III, IV) [28]. The *AJCC*^*8th*^ staging was generated from the T-stage, N-stage, tumour site and HPV status with in-house developed algorithm (eMethods). If HPV status was unknown/unspecified, it was assumed as HPV-negative for non-OPC cases. Categorisation was determined on the Kaplan–Meier curves in the training data to meet adequate proportionality testing (eFig. 1).

### 2.4. Statistical analysis

The MDACC dataset was split into a training and independent validation cohort for the clinical model development (Fig. 1B). The data with all variables collected (i.e., complete cases) were split with a 60:40 ratio into training:validation data. Cases with missing variables (i.e., partial cases) were added to the training set. Only complete cases were considered for the independent and external validation cohorts.

Step-wise forward variable selection was employed to select variables for the Cox regression OS, LC, and RC model based on likelihood ratio test with a Bonferroni-corrected significance level of p < 0.005. Repeated selection was performed on 10 imputed datasets using Multivariate Imputation by Chained Equations (R-package ‘mice’ v3.13.0) with predictive mean matching across 25 iterations [29]. Based on the variable selection and intervariable correlation results, potential models were tested in the validation cohorts. The final models were used for patient stratification. The final OS model was compared with a model based on *AJCC*^*8th*^ alone with the likelihood ratio test.

### 2.5. Risk-based patient stratification

Patients were stratified into high-, intermediate-, and low-risk groups based on the predicted 2-year mortality risk derived from the Cox regression clinical models. These 2-year mortality risk thresholds were visually determined in the training cohort by evaluating the Kaplan–Meier curves for the different risk groups.

### 2.6. Imaging prediction component

For a subset of patients with available pre-treatment contrast-enhanced CT scans, image characteristics of the primary tumour were quantified in geometric and texture radiomics features using previously developed libraries [30,31], according to the Image Biomarker Standardisation Initiative [32]. Features were selected with bootstrapped forward stepwise variable selection (1000 samples). Subsequently, model improvement was tested for the addition of these features to the clinical risk prediction (i.e., linear predictor).

## 3. Results

### 3.1. Patients

A total of 4611 HNC patients were used for the analyses: training (MDACC; n = 2241), independent test (MDACC; n = 786), external validation cohort 1 (UMCG; n = 1087), and external validation cohort 2 (PMH; n = 497). Patient characteristics per cohort are shown in Table 1. Noteworthy differences between cohorts were seen in HPV status (ranging from 16 to 71%), OPC incidence (30–100%), and pack-years (μ = 20–31). Imputation of clinical variables was only performed in the training cohort for *pack-years* (5% missing), *performance score* (16%), and *HPV status* (19%). The overall median follow-up time was 3.6 year (interquartile range [IQR]: [1.6–6.0]), and for censored patients (i.e. excluding patients that die) only 4.3 year [IQR: 2.1–6.7] (site specific, MDACC: 4.1 [2.1–6.6], UMCG: 3.2 [1.7–5.1], and PMH: 8.0 [6.1–9.3]).

### 3.2. Association of clinical variables and treatment outcome

For OS, univariable analyses showed that all clinical variables were significant (p < 0.0001), except *gender* (eTable 1). For LC or RC, all variables were significant, except *age* and *gender* (p > 0.106), and *N-stage* for LC (p = 0.189).

For comprehensive multivariable model analyses and iterations, please refer to eResults 1.

For OS, the final model included the following clinicodemographic variables: *performance score*, *AJCC*^*8th*^ *stage*, *pack-years*, and *age* (Table 2); note that *AJCC*^*8th*^ *stage* is based on *T*- and *N-stage*, *tumour site*, and *HPV-status*. The performance of the OS clinical model was good in both the MDACC training (c-index = 0.72 95% CI [0.66–0.77]) and independent validation cohort (c-index = 0.76 [0.69–0.83]). External validation showed good performance in both the UMCG cohort (c-index = 0.73 [0.68–0.77]) and PMH cohort (c-index = 0.75 [0.68–0.80]). *AJCC*^*8th*^ staging alone was significantly inferior (p < 0.0001) to clinical OS model with c-indices: training 0.65 [0.59–0.71]; test 0.72[0.64–0.80]; UMCG 0.67 [0.62–0.72]; PMH 0.69 [0.62–0.76].

The final LC model contained *T-stage*, *HPV status*, *performance score*, and *pack-years*, with resultant c-indices: training: 0.74 [0.70–0.78]; testing: 0.71 [0.58–0.84]; external validation: 0.70 [0.62–0.76] (UMCG); and 0.74 [0.59–0.89) (PMH). T-stage (HR: T2, 4.19 [2.19–8.03]); T3, 4.36 [2.22–8.58]; T4, 5.02 [2.56–9.83]) and HPV status (HR: 0.5 [0.34–0.73]) were the most dominant factors in predicting LC.

The final RC model included *AJCC*^*8th*^ *stage*, *tumour site*, and *performance score* as component variables (Table 2). Resultant c-indices showed training: 0.74 [0.69–0.78]; testing: 0.73 [0.57–0.89]; external validation: 0.7 [0.62–0.77] (UMCG) and 0.71 [0.48–0.94] (PMH). While N-stage can be expected to be an important predictor for RC, the combination of tumour characteristics in the *AJCC*^*8th*^ outperformed N-stage alone.

Overall, the calibration plots and HosmereLemeshow analyses showed good calibration of the models in the comparator cohort (eFig. 2). Yet, significant calibration deviation was seen for the OS model in the external cohorts.

### 3.3. Model-based patient stratification

**Fig. 2.**
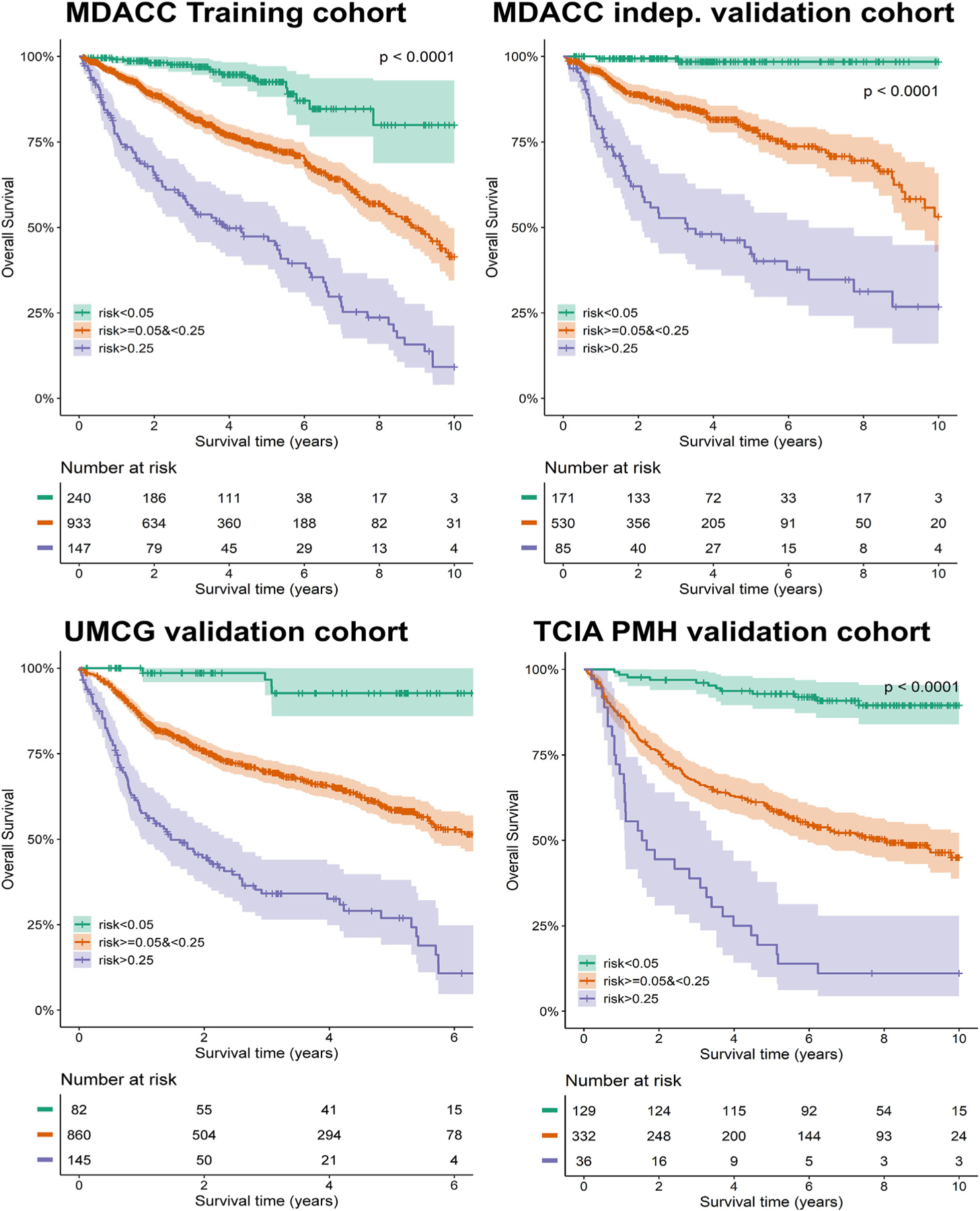
Patient stratification based on predicted mortality risk. Survival curves for low risk (in green; 2-year mortality risk<5%), intermediate risk (in orange; risk≥5 and <25%), and high risk (in blue; ≥25%) in training, validation, and two external validation cohort. Note: Follow-up time was truncated at 6 years for UMCG and 10 years for MDACC and PMH data. (For interpretation of the references to colour in this figure legend, the reader is referred to the Web version of this article.)

The survival curves of patients stratified based on their model-based predicted 2-year mortality risk (2y-risk) are shown in Fig. 2. Based on the training cohort, the best separation was seen for predicted 2y-risk lower than 5% (low-risk), between 5 and 25% (intermediate-risk), and higher than 25% (high risk). The average observed 5-year OS was 95% (range: 93–98%) for the low-risk group, 65% (58–79%) for the intermediate-risk group, and 29% (17–42%) for the high-risk group. Notably, the proportion of MDACC and PMH patients stratified as low risk (20% and 26%) was substantially larger compared to the UMCG patients (8%). See eFigures R1.2 and R1.3 for LC and RC analyses.

**Fig. 3.**
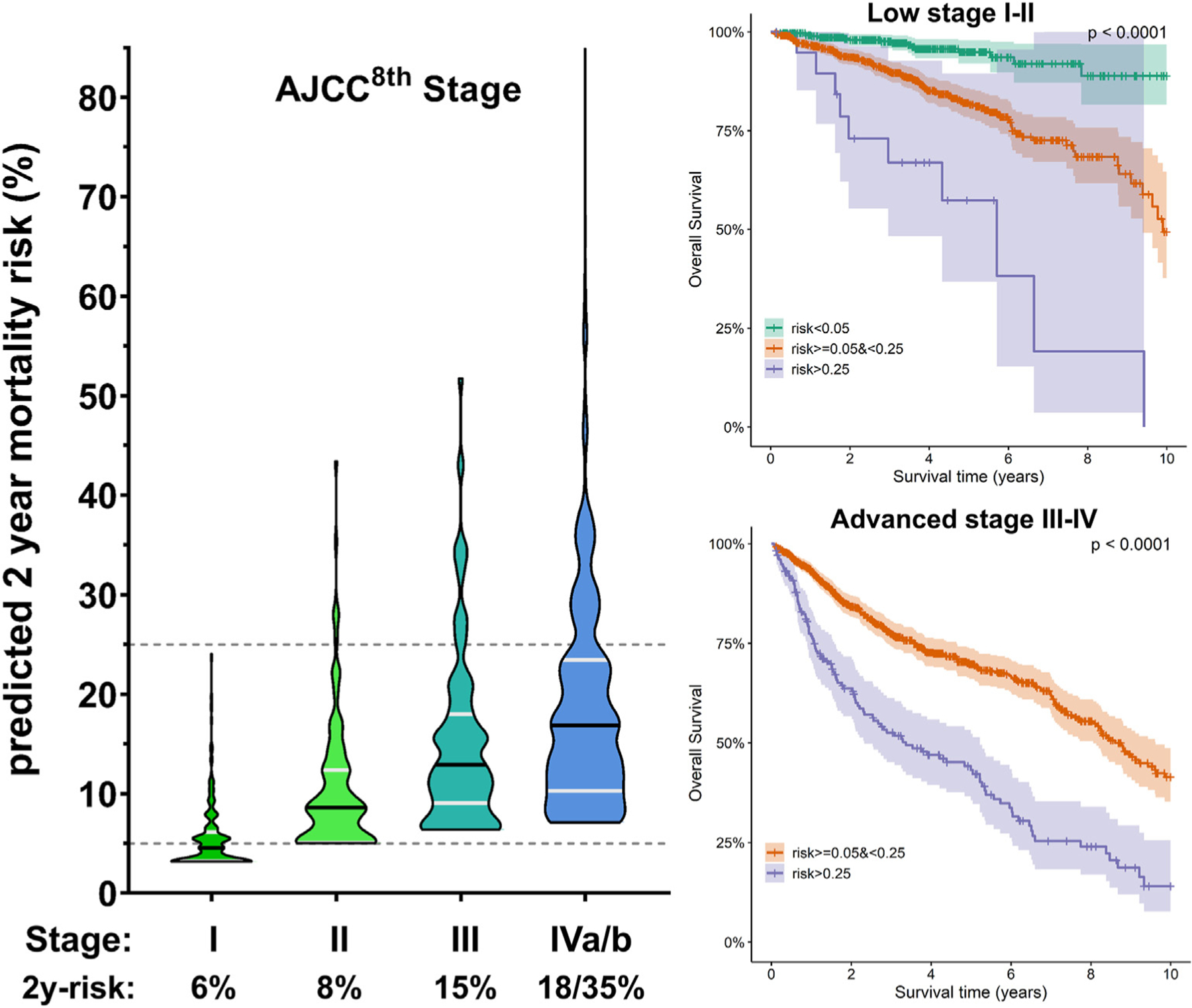
Predicted overall survival risk based on clinical model versus *AJCC*^*8th*^ staging. Predicted 2-year mortality risk (y-axis) depicted per *AJCC*^*8th*^ stage group (left); percentages on x-axis are risks predicted based on staging alone. Survival curves show clear split with model-based risk stratification both in patients with low (right, top) and advanced *AJCC*^*8th*^ stage (right, bottom) patients. These figures are based on the MDACC data.

Prediction based on AJCC^8th^ staging alone gives a single 2y-risk per category (x-axis Fig. 3-left), while a sizeable spread can be seen per category in 2y-risk calculated by the clinical model (y-axis). Fig. 3 shows that only a select portion of the Stage I is low risk (2y-risk<5%), and limited number of Stage III-IV patients are high risk (2y-risk>25%). The ‘by-the-model-identified’ high-risk patients were correctly classified as the majority of these patients died (Fig. 3-right).

### 3.4. Web interface prediction and stratification tool

The clinically usable prediction tool was implemented in an interactive web interface https://uic-evl.github.io/hnc-predictor/employing the final clinical models. Here, the clinical variables of a new patient (e.g., age) can be interactively submitted, whereafter the patient-specific predicted OS, LC, or RC curves can be calculated. Finally, by submitting the desired 2-y risk threshold, the new patient is stratified into being low, intermediate, high risk of OS, LC, and/or RC.

### 3.5. Models in tumour site sub cohorts

The clinical models performed well in two largest sub-cohorts: OPC (n = 2930 patients) and larynx (n = 1257) with c-indices of 0.77/0.76/0.71 and 0.70/0.63/0.73 for OS/LC/RC, respectively (eFig. 3). The model performance (c-index: 0.66/0.67/0.64) was lower for the oral cavity patients (n = 805). Overall, the calibration of the models was good, yet the actual mortality risk was higher than predicted for the OPC and oral cavity patients (HosmereLemeshow p-value<0.05), which was comparable to the total cohort. The number of hypopharynx (n = 136), nasopharynx (n = 56), and unknown primary (n = 73) patients was too low to draw reliable conclusions (eFig. 3).

### 3.6. Imaging component

For the radiomics features, 455 MDACC patients were used for training, and 229 UMCG and 430 PMH patients for external validation. The bootstrapped stepwise forward selection identified the ‘minor axis length’ of the primary tumour as the most frequently selected geometric predictor for OS (eResults 2). This image feature significantly added (likelihood ratio test; p = 0.004) to predicted risk from clinical model (i.e., linear predictor). Compared to the clinical model (c-index = 0.72 [0.63–0.81]), the performance of this combined model increased slightly (c-index 0.73 [0.64–0.81]). While the validation c-index increase was more pronounced in the UMCG cohort (from 0.71 [0.62–0.81] to 0.74 [0.64–0.83]), no performance improvement was seen in the PMH validation cohort (from 0.74 [0.67–0.80] to 0.74 [0.67–0.81]). No robust features could be identified for LC and RC (eResults 2).

## 4. Discussion

The clear stratification of patients with HNC into high, intermediate, and low risk of mortality (Fig. 2) by the models can be effectively used for personalised radiotherapy, e.g., selecting high-risk patients for tumour radiation dose escalation or low-risk patients for dose de-escalation. The impressive survival differences for patients who are nominally in the same AJCC (including HPV) risk category allows for more directive and granular patient-by-patient risk differentiation. For example, OPC HPV-positive patients are considered for de-escalation trials [13–15], yet our findings show that 4% and 14% of these patients have a 2-y mortality of >25% and >15%, respectively, for which dose de-escalation may not be advisable. By using this international big dataset of more than 4500 patients, this study establishes a benchmark for robust OS, LC, and RC prediction in patients with HNC. Additionally, the clinic-ready web-based tool calculates and visualises the expected survival and tumour outcome for new individual patients (https://uic-evl.github.io/hnc-predictor/). The underlying model code, radiomics, and clinical data are publicly shared in a Figshare repository: https://doi.org/10.6084/m9.figshare.21303000.

All final clinical models included the patient’s *performance score*; that poor(er) performance scores are associated with poorer survival has been long recognised [33,34], yet that tumour control is associated with performance status is less intuitive. The composite variable *AJCC*^*8th*^ *staging* together with *pack-years*, *age*, and *performance score* were included in the OS model; hence, all clinical variables were directly or indirectly incorporated in this model, except gender. Similar OS risk factors have been observed in previous studies, age, tumour location, smoking status, T and N-stage [20,35], and later HPV status [18,19]. Beesley *et al.* developed a US-trained/EU-validated multistate Bayesian clinical prediction model for radiotherapy OPC patients to predict event likelihood parameters [36]. While the modelling procedure was quite different, similar input predictors were identified: T, N-stage, HPV status, age, smoking status; notably, tobacco pack-years and performance score were not included. Overall, these findings suggest that despite distinct modelling approaches and datasets, convergent phenomena have been observed.

For the LC prediction, *T-stage*, *HPV status*, *performance score*, and *pack-years* were selected. Since *HPV status* was highly correlated to *tumour site* (Rho = 0.89; p < 0.0001), it is difficult to determine the impact of tumour location on LC. In contrast, for RC, tumour site showed added predictive value to *AJCC*^*8th*^ staging, which is interesting as it based on the tumour site. This is likely due to the difference of the lymphatic tumour spread per tumour location [37].

Outcome prediction was robust across multi-institutional cohorts, even though they had distinct patient demographic profiles (Table 1); particularly, the HPV-positive HNC incidence was substantially lower in European compared to the North American cohorts. Additionally, OPC, larynx, and oral cavity cancer sub-analyses (eFig. 3) showed clinical applicable levels of model performance and calibration. For the hypopharynx, nasopharynx, and unknown primary cancer sub-cohorts, caution is advised when applying these models due to the sparse patient numbers.

The serial approach of building the prediction model presented in this study (Fig. 1A) allows for flexible addition of imaging features. Higher OS risk was associated with larger *minor axis length* of the tumour [32], which represents an intuitive metric for tumour size. Previous studies showed the relation between OS and features indicating larger or more irregular tumours [22,23]. Texture features, in contrast to prior works [22–24], failed to improve our model discrimination (eResults 2.2); similar to a previous study [38]. This may be due to the sensitivity of intensity/texture features to image acquisition discrepancies [39], arguing for improved image harmonisation, standardisation, and image quality.

Limitations of the study cohort are that the majority of tumour locations were OPC, larynx, and oral cavity, underrepresenting hypopharynx, nasopharynx, and unknown primary cases. While this is a representative of the HNC clinical incidence, this may mean that the presented models are not sufficiently tested for under-represented tumour sites. Another challenge is the definition of local and RC, for which an event was broadly defined as recurrent, progressive, or residual disease. The detection residual/returning disease can be challenging [40] and is further complicated when no salvage treatments are available or when patients are lost from follow-up, and thus, no pathologic conformation, clinical progression, or imaging can be obtained. This may therefore potentially result in an underdetection bias of disease control in the cohorts, which can influence accuracy of the LC and RC models.

As with multi-site data aggregation and risk modelling efforts at large scale, there are intrinsic limitations as function of data availability, e.g., anaemia identified by Beesley *et al.* was not recorded in these datasets [36]. Consequently, the utility of this (or any) predictive model is necessarily predicated on input variables and could be modified or altered with updated or augmented data. Moreover, stage migration considerations between AJCC 7th and 8th edition should be noted; for example, extranodal extension was not always specified/recorded as a formalised component of AJCC 7th ed. and may have been obscured. Improved incorporation could improve the models, or alternatively, it could be added as a separate variable [36]. While we focus on OS, LC, and RC, future work will focus on predicting distant metastases and disease-free survival.

Nonetheless, this study is to our knowledge based on the largest head and neck extant multi-site dataset, which allowed for the development of statistically robust and clinic-ready HNC risk models. This provides a benchmark platform for extended future developments of image-incorporating prediction methods, such as deep learning. Moreover, the end-user-enabled web interface (GUI) provides an accessible decision support tool for patient-individual risk stratification for therapeutic selection.

## 5. Conclusion

Developed and assessed in this international ‘big-data’-set, our prediction models presented excellent capacity to stratify patients with HNC at high, intermediate, and low mortality risk – outperforming *AJCC*^*8th*^ staging. This work sets a benchmark for robust OS, LC, and RC risk prediction in radiotherapy HNC patients, which can effectively be capitalised for personalised radiotherapy with the clinic-ready web-based tool prediction tool for new patients that does not require under-the-hood knowledge of model mechanics (https://uic-evl.github.io/hnc-predictor/).

## Supplementary Material

Appendix A. Supplementary data

## Figures and Tables

**Table 1:** Demographics for training, independent validation, and two validation cohorts.

	MDACC training		MDACC validation		UMCG validation		TCIA PMH validation		p-value
**N**	2241		786		1087		497		
**Age (mean (SD))**	59.48	(10.14)	59.74	(9.74)	64.17	(10.56)	60.16	(9.90)	<0.001
**Sex (%)**									
Female	373	(17)	130	(17)	324	(30)	105	(21)	<0.001
Male	1868	(83)	656	(83)	763	(70)	392	(79)	
**T stage (%)**									<0.001
T0	53	(2)	17	(2)	2	(0)	0	(0)	
T1	507	(23)	178	(23)	196	(18)	90	(18)	
T2	788	(35)	288	(37)	262	(24)	162	(33)	
T3	453	(20)	162	(21)	251	(23)	146	(29)	
T4	414	(18)	136	(17)	376	(35)	99	(20)	
Tx	26	(1)	5	(1)	0	(0)	0	(0)	
**N stage (%)**									<0.001
N0	487	(22)	152	(19)	435	(40)	82	(16)	
N1	276	(12)	116	(15)	130	(12)	48	(10)	
N2a-b	1081	(48)	356	(45)	279	(26)	202	(41)	
N2c	318	(14)	141	(18)	202	(19)	123	(25)	
N3	79	(4)	21	(3)	37	(3)	42	(8)	
**HPV status (%)**									<0.001
Negative	617	(28)	288	(37)	912	(84)	142	(29)	
Positive	990	(44)	498	(63)	175	(16)	355	(71)	
Unknown	634	(28)	0	(0)	0	(0)	0	(0)	
**Site (%)**									<0.001
Oropharynx	1382	(62)	462	(59)	328	(30)	497	(100)	
Larynx	420	(19)	179	(23)	446	(41)	0	(0)	
Oral Cavity	314	(14)	95	(12)	263	(24)	0	(0)	
Hypopharynx	50	(2)	32	(4)	26	(2)	0	(0)	
Nasopharynx	22	(1)	0	(0)	23	(2)	0	(0)	
Unkown primary	53	(2)	18	(2)	1	(0)	0	(0)	
**AJCC**^**8th**^ **stage (%)**									<0.001
I	605	(27)	271	(34)	159	(15)	156	(31)	
II	368	(16)	157	(20)	163	(15)	137	(28)	
III	349	(16)	143	(18)	247	(23)	106	(21)	
IVa	472	(21)	206	(26)	491	(45)	87	(18)	
IVb	29	(1)	9	(1)	27	(2)	11	(2)	
Unknown	418	(19)	0	(0)	0	(0)	0	(0)	
**Performance score (%)**									<0.001
0	850	(38)	397	(51)	619	(57)	323	(65)	
1	620	(28)	319	(41)	350	(32)	125	(25)	
>2	181	(8)	70	(9)	118	(11)	49	(10)	
Unknown	590	(26)	0	(0)	0	(0)	0	(0)	
**Smoking status (%)**									<0.001
Never	773	(34)	301	(38)	175	(16)	144	(29)	
Former	998	(45)	360	(46)	457	(42)	198	(40)	
Current	453	(20)	125	(16)	427	(39)	155	(31)	
Unknown	17	(1)	0	(0)	28	(3)	0	(0)	
**Pack years (mean (SD))**	22.03	(33.69)	20.01	(28.19)	30.77	(23.90)	24.35	(24.67)	<0.001
**Chemotherapy (%)**									<0.001
None	446	(20)	115	(15)	696	(64)	254	(51)	
Concurrent	1060	(47)	410	(52)	389	(36)	243	(49)	
Induction	218	(10)	100	(13)	1	(0)	0	(0)	
Induction + concurrent	480	(21)	161	(20)	1	(0)	0	(0)	
Unknown	37	(2)	0	(0)	0	(0)	0	(0)	
**Technique (%)**									<0.001
3DCRT	211	(9)	9	(1)	14	(1)	0	(0)	
IMRT	1496	(67)	450	(57)	517	(48)	497	(100)	
VMAT	466	(21)	292	(37)	401	(37)	0	(0)	
IMPT	68	(3)	35	(4)	111	(10)	0	(0)	
Unknown	0	(0)	0	(0)	44	(4)	0	(0)	
**Radiotherapy type (%)**									<0.001
Primary	1727	(77)	644	(82)	852	(78)	497	(100)	
Post-operative	251	(11)	40	(5)	230	(21)	0	(0)	
Unknown	263	(12)	102	(13)	5	(0)	0	(0)	
**Mortality events (%)**	635	(28)	148	(19)	402	(37)	206	(41)	<0.001
**Local failure events (%)**	233	(10)	70	(9)	149	(14)	46	(9)	<0.001
**Regional failure events (%)**	182	(8)	48	(6)	105	(10)	31	(6)	0.005

Abbreviations: SD: standard deviation; HPV, human papilloma virus; 3DCRT, three-dimensional conformal radiotherapy; IMRT, intensity-modulated radiotherapy; VMAT, volumetric-modulated arc therapy; IMPT, intensity-modulated proton therapy.

**Table 2:** Clinical model parameters and c-index model performance.

Overall survival (OS)				
Variables	Category	Coefficients	Hazard ratio	p value
Performance score	0	0	1	ref
	1	0.469	1.6 (1.28–1.99)	<0.0001
	≥2	0.781	2.18 (1.51–3.16)	0.0001
*AJCC*^*8th*^ stage	I	0	1	ref
	II	0.117	1.12 (0.76–1.65)	0.5545
	III	0.679	1.97 (1.42–2.74)	0.0001
	IVa	0.793	2.21 (1.66–2.94)	<0.0001
	IVb	1.509	4.52 (2.79–7.33)	<0.0001
Pack years	≤5	0	1	ref
	5–25	0.267	1.31 (1.01–1.7)	0.0459
	26–50	0.499	1.65 (1.3–2.08)	<0.0001
	>50	0.867	2.38 (1.78–3.17)	<0.0001
Age	≤55	0	1	ref
	56–65	0.085	1.09 (0.89–1.33)	0.4113
	65–75	0.400	1.49 (1.2–1.85)	0.0003
	>75	0.753	2.12 (1.56–2.89)	<0.0001
Local control (LC)				
Variables	Category	Coefficients	Hazard ratio	p value

T stage	T1	0	1	ref
	T2	1.432	4.19 (2.19–8.03)	<0.0001
	T3	1.473	4.36 (2.22–8.58)	<0.0001
	T4	1.613	5.02 (2.56–9.83)	<0.0001
HPV status	positive = 1	−0.694	0.5 (0.34–0.73)	0.0003
Performance score	0	0	1	ref
	1	0.421	1.52 (1.05–2.22)	0.0276
	≥2	0.801	2.23 (1.38–3.59)	0.0010
Pack years	≤5	0	1	ref
	5–25	−0.039	0.96 (0.58–1.6)	0.8807
	26–50	0.294	1.34 (0.87–2.08)	0.1858
	>50	0.496	1.64 (1.02–2.64)	0.0403
Regional control (RC)				
Variables	Category	Coefficients	Hazard ratio	p value

AJCC8th stage	I	0	1	ref
	II	0.442	1.56 (0.7–3.46)	0.2774
	III	0.984	2.68 (1.28–5.59)	0.0089
	IVa	1.567	4.79 (2.34–9.81)	<0.0001
	IVb	2.565	13 (4.76–35.55)	<0.0001
Performance score	0	0	1	ref
	1	0.573	1.77 (1.15–2.73)	0.0093
	≥2	0.793	2.21 (1.27–3.84)	0.0049
Tumour site	Hypopharynx	0	1	ref
	Larynx	−0.118	0.89 (0.45–1.75)	0.7343
	Oropharynx	−0.648	0.52 (0.25–1.11)	0.0898
	Oral cavity	−0.853	0.43 (0.21–0.88)	0.0203
	Unknown Prim	−1.140	0.32 (0.07–1.51)	0.1493
	Nasopharynx	−4.995	0.01 (0–21498.48)	0.9932
Model performance (c-index [95%CI])				
	MDACC Training	MDACC validation	UMCG external validation 1	MGH external validation 2

Overall Survival (OS)	0.72 [0.66–0.78]	0.76 [0.68–0.83]	0.73 [0.68–0.78]	0.75 [0.69–0.81]
Local control (LC)	0.74 [0.67–0.82]	0.71 [0.58–0.84]	0.70 [0.62–0.77]	0.75 [0.61–0.90]
Regional control (RC)	0.74 [0.64–0.83]	0.73 [0.57–0.89]	0.7 [0.62–0.78]	0.74 [0.56–0.91]

Abbreviations: HPV, human papilloma virus; CI, confidence interval.
