# Supplementary material for: Head and neck cancer predictive risk estimator to determine control and therapeutic outcomes of radiotherapy (HNC-PREDICTOR): development, international multi-institutional validation, and web implementation of clinic-ready model-based risk stratification for head and neck cancer
Source: Eur J Cancer. Author manuscript; Available in PMC 2023 Jan 20. (PMC9853413; doi:10.1016/j.ejca.2022.10.011)
Supplement: Appendix A. Supplementary data [file NIHMS1864360-supplement-Appendix_A__Supplementary_data.docx]

**Supplementary data**

**Table of contents**

| **eMethods** | R script stage_maker – generating the AJCC^8th^ variable |
| --- | --- |
| **eFigure 1** | Kaplan Meyer curves for Overall Survival |
| **eTable 1** | Univariable analyses |
| **eTable 2** | Correlation between clinical variables |
| **eTable 3** | HPV status per tumor site |
|  |  |
| **eResults 1** | **Clinical variable selection** |
| **eResults 1.1** | Summary of variable selection and modelling considerations for Overall Survival (OS) |
|  | eTable R1.1.1 Forward selection in the different imputation sets  eTable R1.1.2 Frequency of selected variables  eTable R1.1.3 Forward step-wise variable selection in complete cases  eTable R1.1.4 Performance (c-index) of potential OS models  eTable R1.1.5 Coefficients (β), Hazard ratio (HR) and p-values of potential OS models |
| **eResults 1.2** | Summary of variable selection and modelling considerations for Local Control (LC) |
|  | eTable R1.2.1 Forward selection in the different imputation  eTable R1.2.2 Frequency of selected variables  eTable R1.2.3 Forward step-wise variable selection in complete cases  eTable R1.2.4 Performance (c-index) of potential LC models  eTable R1.2.5 Coefficients (β), Hazard ratio (HR) and p-values of potential LC models |
|  | eFigure R1.2 Patient stratification plots for local control risk |
| **eResults 1.2** | Summary of variable selection and modelling considerations for Regional Control (RC) |
|  | eTable R1.3.1 Forward selection in the different imputation sets  eTable R1.3.2 Frequency of selected variables  eTable R1.3.3 Forward step-wise variable selection in complete cases  eTable R1.3.4 Performance (c-index) of potential RC models  eTable R1.3.5 Coefficients (β), Hazard ratio (HR) and p-values of potential RC models |
|  | eFigure R1.3 Patient stratification plots for regional control risk |
|  |  |
| **eFigure 2** | Calibration plot for the clinical prediction models for the different cohorts |
| **eFigure 3** | Calibration plot of the clinical prediction models for the different tumor site |
|  |  |
| **eResults 2** | **Image biomarker selection** |
| ***eResults 2.1*** | Summary of variable selection and modelling considerations for Geometric features |
|  | eFigure R2.1. Frequency plot of image variables  eTable R2.1.1. Univariable analyses of geometric features  eTable R2.1.2. Forward step-wise variable selection geometric  eTable R2.1.3 Potential clinical + geometric radiomics models |
| **eResults 2.2** | Summary of variable selection and modelling considerations for Texture features |
|  | eFigure R2.2.1 Frequency plot of image variables  eTable R2.2.1 Univariable analyses of texture features  eTable R2.2.2 Forward step-wise variable selection for Texture features  eTable R2.2.3 Potential clinical + geometric +texture radiomics models |

**eMethods. R script stage_maker – generating the AJCC^8th^ variable**

stage_maker<-function(DATA){

DATA$stage_new="fill"

ind_OPpos=(DATA$site == "OPC" & DATA$HPV.P16.status=="Positive")

DATA_temp=DATA[ind_OPpos,]

DATA_temp$stage_new[DATA_temp$T_stage %in% c("T0","T1","T2","Tx") & DATA_temp$N_stage %in% c("N0","N1","N2a-b")] ="I"

DATA_temp$stage_new[DATA_temp$T_stage %in% c("T0","T1","T2","Tx") & DATA_temp$N_stage %in% c("N2c")] ="II"

DATA_temp$stage_new[DATA_temp$T_stage %in% c("T3") & DATA_temp$N_stage %in% c("N0","N1","N2a-b","N2c")]="II"

DATA_temp$stage_new[DATA_temp$T_stage %in% c("T4") | DATA_temp$N_stage %in% c("N3")] ="III"

DATA_temp[DATA_temp$stage_new=="fill",c("T_stage","N_stage")]

DATA$stage_new[ind_OPpos]=DATA_temp$stage_new

ind_OPneg=(DATA$site == "OPC" & DATA$HPV.P16.status=="Negative")|DATA$site %in% c("Oral Cavity","Hypopharynx","Larynx")

DATA_temp=DATA[ind_OPneg,]

DATA_temp$stage_new[DATA_temp$T_stage %in% c("T0","Tx","T1") & DATA_temp$N_stage %in% c("N0")] ="I"

DATA_temp$stage_new[DATA_temp$T_stage %in% c("T2") & DATA_temp$N_stage %in% c("N0")] ="II"

DATA_temp$stage_new[DATA_temp$T_stage %in% c("T1","T2","Tx") & DATA_temp$N_stage %in% c("N1")] ="III"

DATA_temp$stage_new[DATA_temp$T_stage %in% c("T3") & DATA_temp$N_stage %in% c("N0","N1")] ="III"

DATA_temp$stage_new[DATA_temp$T_stage %in% c("T4") | DATA_temp$N_stage %in% c("N2a-b","N2c")] ="IVa"

DATA_temp$stage_new[ DATA_temp$N_stage %in% c("N3")] ="IVb"

DATA_temp[DATA_temp$stage_new=="fill",c("T_stage","N_stage")]

DATA$stage_new[ind_OPneg]=DATA_temp$stage_new

ind_NASO=(DATA$site == "Nasopharynx" )

DATA_temp=DATA[ind_NASO,]

DATA_temp$stage_new[DATA_temp$T_stage %in% c("T0","Tx","T1") & DATA_temp$N_stage %in% c("N0")] ="I"

DATA_temp$stage_new[DATA_temp$T_stage %in% c("T0","Tx","T1") & DATA_temp$N_stage %in% c("N1")] ="II"

DATA_temp$stage_new[DATA_temp$T_stage %in% c("T2") & DATA_temp$N_stage %in% c("N0","N1")] ="II"

DATA_temp$stage_new[DATA_temp$T_stage %in% c("T0","Tx","T1") & DATA_temp$N_stage %in% c("N2a-b","N2c")] ="III"

DATA_temp$stage_new[DATA_temp$T_stage %in% c("T2","T3") & DATA_temp$N_stage %in% c("N2a-b","N2c")] ="III"

DATA_temp$stage_new[DATA_temp$T_stage %in% c("T3") & DATA_temp$N_stage %in% c("N0","N1")] ="III"

DATA_temp$stage_new[DATA_temp$T_stage %in% c("T4") | DATA_temp$N_stage %in% c("N3")] ="IVa"

DATA_temp[DATA_temp$stage_new=="fill",c("T_stage","N_stage")]

DATA$stage_new[ind_NASO]=DATA_temp$stage_new

ind_Unk=(DATA$site == "Unkown_primary" )

DATA_temp=DATA[ind_Unk,]

DATA_temp$stage_new[DATA_temp$N_stage %in% c("N1","N2a-b")] ="III"

DATA_temp$stage_new[DATA_temp$N_stage %in% c("N2c")] ="IVa"

DATA_temp$stage_new[DATA_temp$N_stage %in% c("N3")] ="IVb"

DATA_temp[DATA_temp$stage_new=="fill",c("T_stage","N_stage")]

DATA$stage_new[ind_Unk]=DATA_temp$stage_new

DATA$stage_new=factor(DATA$stage_new,levels=c('I','II','II','III','IVa','IVb'),

labels=c('I','II','II','III','IVa','IVb'))

DATA$stage_new_7th=DATA$stage_new

ind_OPpos=(DATA$site == "OPC" )

DATA_temp=DATA[ind_OPpos,]

DATA_temp$stage_new_7th[DATA_temp$T_stage %in% c("T0","Tx","T1") & DATA_temp$N_stage %in% c("N0")] ="I"

DATA_temp$stage_new_7th[DATA_temp$T_stage %in% c("T2") & DATA_temp$N_stage %in% c("N0")] ="II"

DATA_temp$stage_new_7th[DATA_temp$T_stage %in% c("T1","T2") & DATA_temp$N_stage %in% c("N1")] ="III"

DATA_temp$stage_new_7th[DATA_temp$T_stage %in% c("T3") & DATA_temp$N_stage %in% c("N0","N1")] ="III"

DATA_temp$stage_new_7th[DATA_temp$T_stage %in% c("T4") | DATA_temp$N_stage %in% c("N2a-b","N2c")] ="IVa"

DATA_temp$stage_new_7th[ DATA_temp$N_stage %in% c("N3")] ="IVb"

DATA_temp[DATA_temp$stage_new_7th=="fill",c("T_stage","N_stage")]

DATA$stage_new_7th[ind_OPpos]=DATA_temp$stage_new_7th

# DATA[DATA$stage_new=="fill",c("site","T_stage","N_stage")]

return(DATA)

}

**eFigure 1. Kaplan Meyer curves for Overall Survival**

**
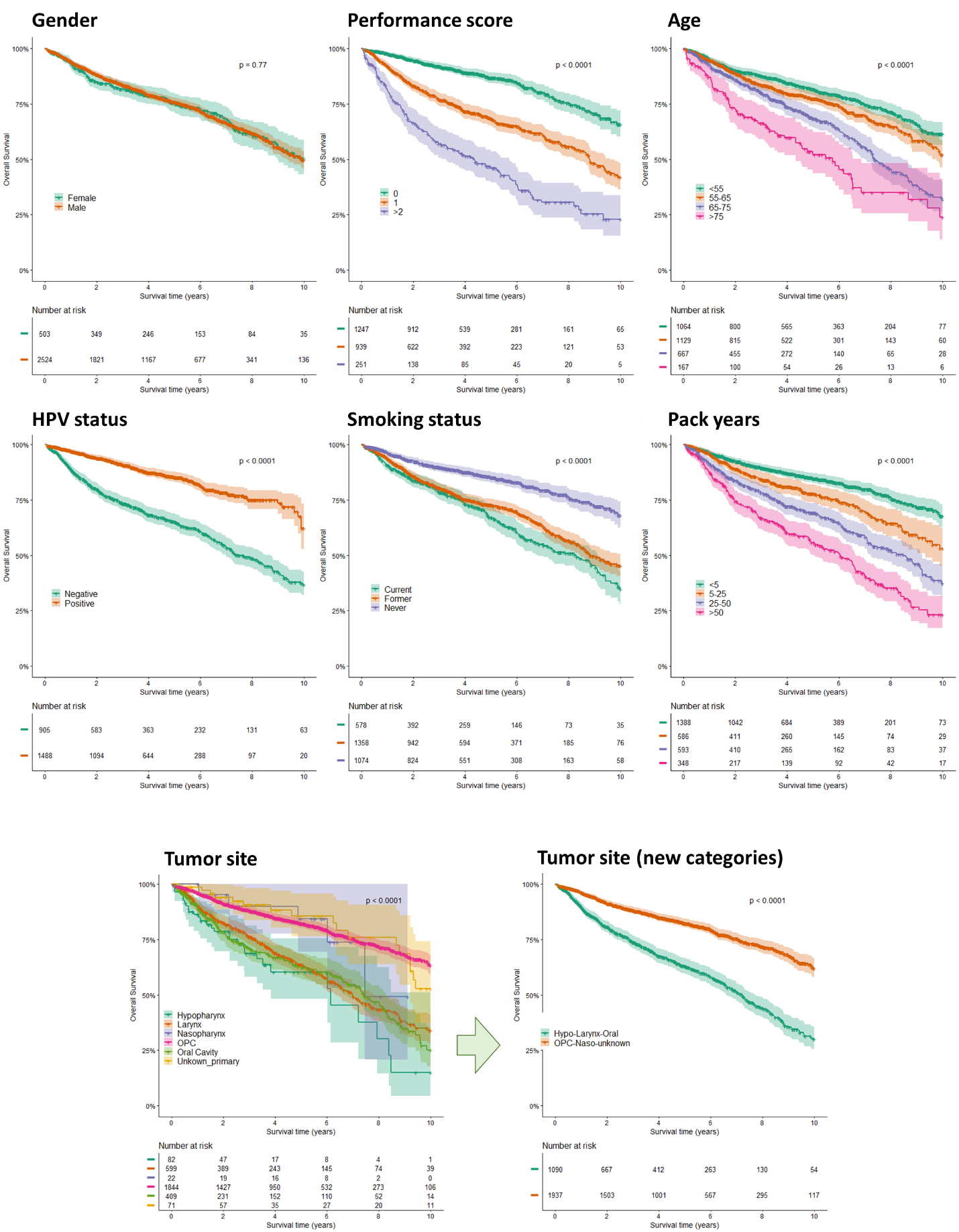
**

**
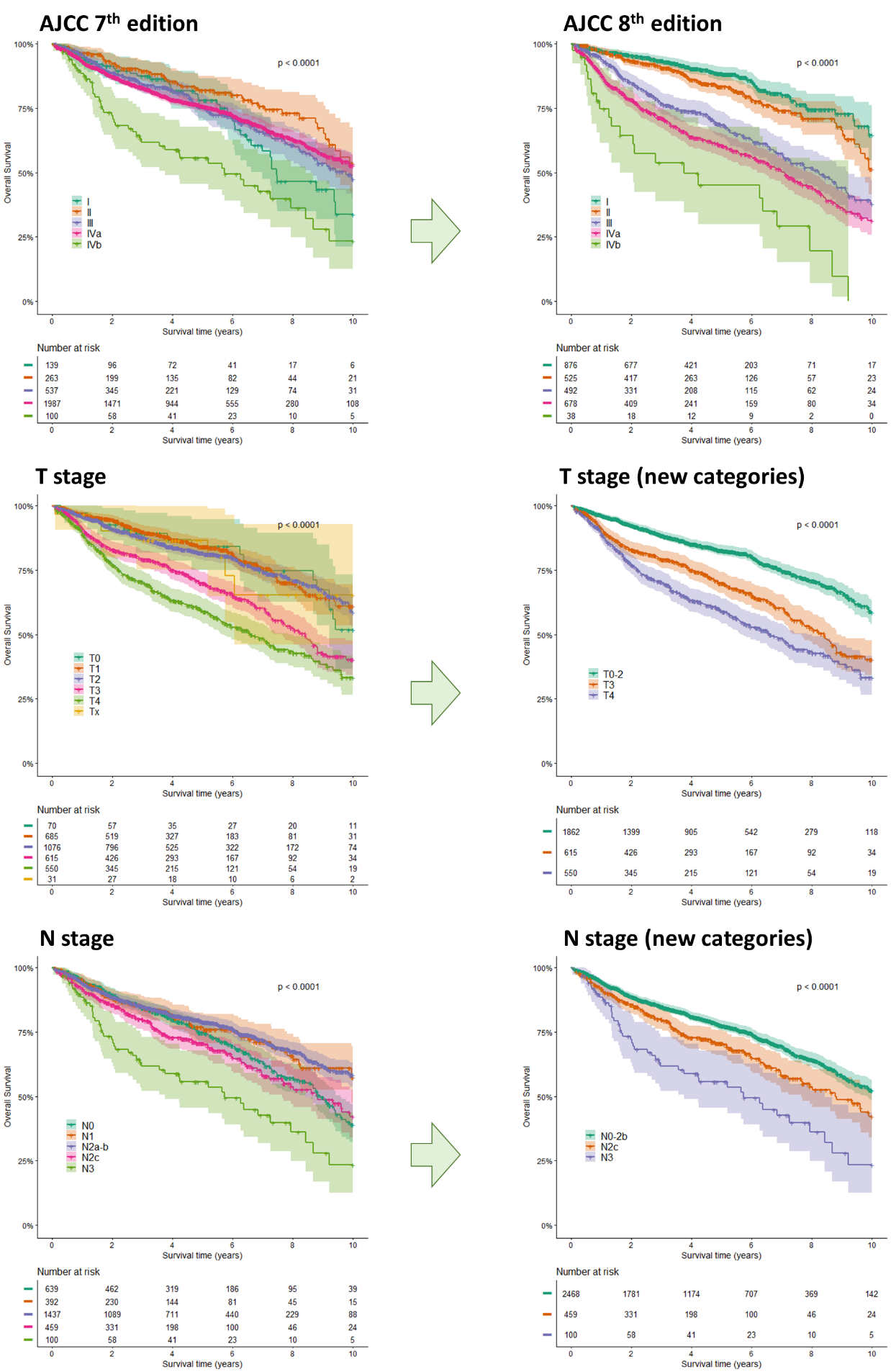
**

**eTable 1. Univariable analyses**

These results are based on the original, non-imputed data based on numeric values.

**eTable 1.1**

| **Overall survival** |  |  |  |
| --- | --- | --- | --- |
|  | **β** | **HR (95% CI)** | **p-value** |
| **Performance score** | **0.82** | **2.26 (1.99-2.57)** | **<0.0001** |
| **pack years** | **0.42** | **1.52 (1.42-1.64)** | **<0.0001** |
| **stage new** | **0.41** | **1.51 (1.4-1.62)** | **<0.0001** |
| **site** | **-0.82** | **0.44 (0.38-0.52)** | **<0.0001** |
| **T stage LC** | **0.37** | **1.45 (1.34-1.56)** | **<0.0001** |
| **HPV status** | **-0.88** | **0.41 (0.34-0.5)** | **<0.0001** |
| **Age** | **0.36** | **1.44 (1.32-1.56)** | **<0.0001** |
| **Smoking status** | **0.70** | **2.02 (1.68-2.44)** | **<0.0001** |
| **N stage** | **0.42** | **1.52 (1.33-1.75)** | **<0.0001** |
| Gender | 0.01 | 1.01 (0.82-1.24) | 0.914 |

**eTable 1.2**

| **Local control** |  |  |  |
| --- | --- | --- | --- |
|  | **β** | **HR (95% CI)** | **p-value** |
| **T stage LC** | **0.44** | **1.55 (1.38-1.75)** | **<0.0001** |
| **stage new** | **0.41** | **1.51 (1.34-1.7)** | **<0.0001** |
| **site** | **-0.91** | **0.4 (0.31-0.52)** | **<0.0001** |
| **HPV status** | **-0.87** | **0.42 (0.31-0.57)** | **<0.0001** |
| **Performance score** | **0.59** | **1.8 (1.47-2.21)** | **<0.0001** |
| **pack years** | **0.32** | **1.38 (1.23-1.54)** | **<0.0001** |
| **Smoking status** | **0.56** | **1.76 (1.31-2.36)** | **<0.0001** |
| Age | 0.12 | 1.13 (0.98-1.31) | 0.097 |
| Gender | -0.26 | 0.77 (0.56-1.06) | 0.125 |
| N stage | 0.19 | 1.21 (0.94-1.55) | 0.153 |

**eTable 1.3**

| **Regional control** |  |  |  |
| --- | --- | --- | --- |
|  | **β** | **HR (95% CI)** | **p-value** |
| **stage new** | **0.57** | **1.77 (1.53-2.06)** | **<0.0001** |
| **N stage** | **0.90** | **2.45 (2-3.01)** | **<0.0001** |
| **Performance score** | **0.67** | **1.95 (1.56-2.44)** | **<0.0001** |
| **T stage LC** | **0.31** | **1.37 (1.19-1.57)** | **<0.0001** |
| **pack years** | **0.28** | **1.33 (1.17-1.51)** | **<0.0001** |
| **site** | **-0.63** | **0.53 (0.4-0.71)** | **<0.0001** |
| **HPV status** | **-0.46** | **0.63 (0.45-0.88)** | **0.007** |
| **Smoking status** | **0.33** | **1.39 (1.01-1.92)** | **0.039** |
| Age | 0.09 | 1.1 (0.93-1.29) | 0.26 |
| Gender | 0.01 | 1.01 (0.68-1.49) | 0.965 |

**eTable 2.** **Correlation between clinical variables**

Polychoric Correlation analyses. Only significant correlations between the clinical variables are depicted. Above the diagonal is the rho of the relation shown, i.e. magnitude of the correlation on scale of 0-1. Below the diagonal, the significant level is shown.

|  | Gender | T_stage | N_stage | HPV.P16.status | site2 | stage_new | Performance_score | AGE | pack_years | Smoking_status |  |
| --- | --- | --- | --- | --- | --- | --- | --- | --- | --- | --- | --- |
| Gender |  |  | 0.022 | 0.282 | 0.237 | -0.133 |  |  |  | 0.028 |  |
| T_stage |  |  | 0.260 |  |  | 0.694 |  |  | 0.180 |  |  |
| N_stage | * | ** |  |  |  | 0.538 |  |  |  |  |  |
| HPV.P16.status | *** |  |  |  | 0.899 | -0.683 |  |  | -0.515 | -0.469 |  |
| site2 | *** |  |  | *** |  | -0.675 |  |  |  | -0.343 |  |
| stage_new | * | *** | *** | *** | *** |  |  |  |  |  |  |
| Performance_score |  |  |  |  |  |  |  | 0.181 |  |  |  |
| AGE |  |  |  |  |  |  | * |  |  |  |  |
| pack_years |  | * |  | ** |  |  |  |  |  | - | 🡪 |
| Smoking_status | *** |  |  | *** | *** |  |  |  | *** |  | Rho |
|  |  |  |  |  |  |  |  |  | 🡨 | p-value |  |

|  | ***=p<0.05** |  |  |  |  |  |  |  |  |  |  |
| --- | --- | --- | --- | --- | --- | --- | --- | --- | --- | --- | --- |
|  | ****=p<0.001** |  |  |  |  |  |  |  |  |  |  |
|  | *****=p<0.0001** |  |  |  |  |  |  |  |  |  |  |
|  |  |  |  |  |  |  |  |  |  |  |  |

**eTable 3. HPV status per tumor site**

|  |  | Tumor site | | | | | |
| --- | --- | --- | --- | --- | --- | --- | --- |
|  |  | Hypopharynx | Larynx | Nasopharynx | OPC | Oral Cavity | Unknown primary |
| HPV status | Negative | 44 | 404 | 22 | 104 | 226 | 33 |
|  | Positive | 6 | 16 | 0 | 860 | 88 | 20 |

**eResults 1. Clinical variable selection**

**eResults 1.1. Summary of variable selection and modelling considerations for Overall Survival (OS)**

The first 4 variables selected in every imputed training dataset were consistently AJCC^8th^ stage, performance score, pack years, and age (eTable R1.1.1). These were identical to those selected if forward step-wise variable selection was performed on the complete training data (i.e., disregarding the patients with missing variables) (eTable R1.1.3).

However, in the imputed datasets additional variables were selected: T stage (10x), tumor site (5x), and N stage (4x) (eTable R1.1.2) The increased data size from 1320 for the complete data to 2241 for the imputed dataset, may explain this increased inclusion of variables, as the p-value is influenced by the data size. The T, N stage and site (together with HPV status), however are the basis of the AJCC^8th^ stage calculation, and therefore inherently correlated. This is also clearly seen in the correlation matrix in eTable 2., demonstrating highly significant (p<0.0001) relations with relatively high correlation magnitude (|rho|>0.54). This dependence also explains why the addition of these variables only show a minimal improvement in c-index performance in the training cohort, and worse performance in validation sets (eTable R1.1.4). Another indication of the multicollinearity of these variables together in a model is the change in model coefficients of the AJCC^8th^ stage eTable R1.1.5, demonstrating the correlation between the variables. The estimation of the coefficients seemed to be slightly better by pooling the coefficients based on the 10 imputation sets, than in the complete data cases, thus the pooled coefficients were used.

In conclusion, the Cox regression model for OS with AJCC^8th^ stage, performance score, pack years and age was selected as the final model.

**eTable R1.1.1 Forward selection in the different imputation sets** for likelihood ratio-test threshold of p<0.005*

*** *Bonferroni corrected p value= 0.05/10*

|  | Variable 1 | Variable 2 | Variable 3 | Variable 4 | Variable 5 | Variable 6 | Variable 7 |  |
| --- | --- | --- | --- | --- | --- | --- | --- | --- |
| Imputation set 1 | pack_years | stage_new | Performance_score | AGE | T_stage | site2 | N_stage |  |
| Imputation set 2 | stage_new | Performance_score | pack_years | AGE | T_stage |  |  |  |
| Imputation set 3 | pack_years | stage_new | Performance_score | AGE | T_stage | N_stage | site2 |  |
| Imputation set 4 | Performance_score | pack_years | stage_new | AGE | site2 | T_stage | N_stage |  |
| Imputation set 5 | pack_years | stage_new | Performance_score | AGE | T_stage | site2 | N_stage |  |
| Imputation set 6 | stage_new | pack_years | Performance_score | AGE | T_stage | N_stage | site2 |  |
| Imputation set 7 | stage_new | pack_years | Performance_score | AGE | T_stage | site2 | N_stage |  |
| Imputation set 8 | Performance_score | stage_new | pack_years | AGE | T_stage | site2 | N_stage |  |
| Imputation set 9 | stage_new | pack_years | Performance_score | AGE | T_stage | N_stage | site2 |  |
| Imputation set 10 | stage_new | pack_years | Performance_score | AGE | T_stage | N_stage | site2 |  |

**eTable R1.1.2 Frequency of selected variables**

|  | **frequency** |
| --- | --- |
| Performance_score | 10 |
| T_stage | 10 |
| HPV.P16.status | 0 |
| stage_new | 10 |
| pack_years | 10 |
| site2 | 5 |
| N_stage | 4 |
| AGE | 10 |
| Gender | 0 |

**eTable R1.1.3 Forward step-wise variable selection in complete cases** (n=1320) based on *likelihood ratio-test* for OS

|  | **Overall Survival** | **(OS)** |  |  |  |  |  |  |  |  |  |
| --- | --- | --- | --- | --- | --- | --- | --- | --- | --- | --- | --- |
|  | ***variables*** | ***categories*** | ***coef*** | ***HR*** | ***p value*** | ***LLH*** | ***ratio-test*** |  | ***AIC*** | ***BIC*** | ***c-index*** |
| **step 1** | **Performance score** | Performance score1 | 0.74 | 2.09 | <0.0001 | -2114 | <0.0001 |  | 4232 | 4240 | 0.66 |
|  |  | Performance score>2 | 1.44 | 4.21 | <0.0001 |  |  |  |  |  |  |
| **step 2** | **Performance score** | Performance score1 | 0.64 | 1.90 | <0.0001 | -2087 | <0.0001 |  | 4185 | 4208 | 0.71 |
|  |  | Performance score>2 | 1.25 | 3.49 | <0.0001 |  |  |  |  |  |  |
|  | **AJCC stage** | stage newII | 0.55 | 1.74 | 0.0053 |  |  |  |  |  |  |
|  |  | stage newIII | 0.85 | 2.33 | <0.0001 |  |  |  |  |  |  |
|  |  | stage newIVa | 0.97 | 2.64 | <0.0001 |  |  |  |  |  |  |
|  |  | stage newIVb | 1.95 | 7.03 | <0.0001 |  |  |  |  |  |  |
| **step 3** | **Performance score** | Performance score1 | 0.57 | 1.76 | <0.0001 | -2075 | <0.0001 |  | 4167 | 4202 | 0.71 |
|  |  | Performance score>2 | 1.15 | 3.17 | <0.0001 |  |  |  |  |  |  |
|  | **AJCC stage** | stage newII | 0.51 | 1.67 | 0.0101 |  |  |  |  |  |  |
|  |  | stage newIII | 0.74 | 2.09 | 0.0001 |  |  |  |  |  |  |
|  |  | stage newIVa | 0.85 | 2.34 | <0.0001 |  |  |  |  |  |  |
|  |  | stage newIVb | 1.79 | 5.98 | <0.0001 |  |  |  |  |  |  |
|  | **Pack years** | pack years5-25 | 0.16 | 1.17 | 0.3430 |  |  |  |  |  |  |
|  |  | pack years25-50 | 0.38 | 1.47 | 0.0084 |  |  |  |  |  |  |
|  |  | pack years>50 | 0.73 | 2.08 | <0.0001 |  |  |  |  |  |  |
| **step 4** | **Performance score** | Performance score1 | 0.56 | 1.75 | <0.0001 | -2067 | 0.0026 |  | 4159 | 4205 | 0.72 |
|  |  | Performance score>2 | 1.11 | 3.04 | <0.0001 |  |  |  |  |  |  |
|  | **AJCC stage** | stage newII | 0.47 | 1.59 | 0.0198 |  |  |  |  |  |  |
|  |  | stage newIII | 0.71 | 2.04 | 0.0001 |  |  |  |  |  |  |
|  |  | stage newIVa | 0.82 | 2.28 | <0.0001 |  |  |  |  |  |  |
|  |  | stage newIVb | 1.74 | 5.70 | <0.0001 |  |  |  |  |  |  |
|  | **Pack years** | pack years5-25 | 0.18 | 1.20 | 0.2757 |  |  |  |  |  |  |
|  |  | pack years25-50 | 0.38 | 1.47 | 0.0085 |  |  |  |  |  |  |
|  |  | pack years>50 | 0.67 | 1.95 | <0.0001 |  |  |  |  |  |  |
|  | **Age** | AGE55-65 | 0.00 | 1.00 | 0.9815 |  |  |  |  |  |  |
|  |  | AGE65-75 | 0.36 | 1.44 | 0.0143 |  |  |  |  |  |  |
|  |  | AGE>75 | 0.62 | 1.87 | 0.0040 |  |  |  |  |  |  |

**eTable R1.1.4. Performance (c-index) of potential OS models** in training and validation cohort

**Model variables:**

**OS Model 1**: Surv ~ Performance_score + stage_new + pack_years + Age

**OS Model 2**: Surv ~ Performance_score + stage_new + pack_years + Age + T_stage

**OS Model 3**: Surv ~ Performance_score + stage_new + pack_years + Age + T_stage + site

**OS Model 4**: Surv ~ Performance_score + stage_new + pack_years + Age + T_stage + site + N_stage

N.b. coefficients in Table D.1.5.

|  |  | **Training** | **Indep. test** | **External val. 1** | **External val. 2** |
| --- | --- | --- | --- | --- | --- |
| **OS model 1** | **complete cases** | 0.72 (0.69-0.75) | 0.76 (0.69-0.83) | 0.73 (0.68-0.77) | 0.75 (0.68-0.8) |
|  | **imputed pooled** | 0.72 (0.66-0.77) | 0.76 (0.68-0.83) | 0.73 (0.68-0.77) | 0.75 (0.69-0.81) |
| **OS model 2** | **complete cases** | 0.72 (0.69-0.75) | 0.76 (0.68-0.83) | 0.73 (0.69-0.78) | 0.74 (0.68-0.8) |
|  | **imputed pooled** | 0.72 (0.66-0.77) | 0.75 (0.67-0.83) | 0.72 (0.67-0.77) | 0.75 (0.69-0.81) |
| **OS model 3** | **complete cases** | 0.73 (0.7-0.76) | 0.76 (0.68-0.83) | 0.72 (0.67-0.76) | 0.74 (0.67-0.79) |
|  | **imputed pooled** | 0.72 (0.66-0.77) | 0.75 (0.67-0.83) | 0.71 (0.67-0.76) | 0.75 (0.69-0.8) |
| **OS model 4** | **complete cases** | 0.73 (0.7-0.75) | 0.76 (0.68-0.83) | 0.72 (0.67-0.77) | 0.73 (0.67-0.8) |
|  | **imputed pooled** | 0.72 (0.66-0.77) | 0.75 (0.67-0.83) | 0.71 (0.66-0.76) | 0.74 (0.68-0.8) |

**eTable R1.1.5 Coefficients (β), Hazard ratio (HR) and p-values of potential OS models**

| **OS model 1** |  |  |  |  |  |  |
| --- | --- | --- | --- | --- | --- | --- |
|  | **β** | **β-pooled** | **HR (95%CI)** | **HR (95%CI)-pooled** | **p-value** | **p-value-pooled** |
| **Performance_score=1** | 0.56 | 0.47 | 1.75 (1.35-2.26) | 1.6 (1.28-1.99) | <0.0001 | <0.0001 |
| **Performance_score=>2** | 1.11 | 0.78 | 3.04 (2.24-4.12) | 2.18 (1.51-3.16) | <0.0001 | 0.0001 |
| **stage_new=II** | 0.47 | 0.12 | 1.59 (1.08-2.36) | 1.12 (0.76-1.65) | 0.0198 | 0.5545 |
| **stage_new=III** | 0.71 | 0.68 | 2.04 (1.42-2.93) | 1.97 (1.42-2.74) | 0.0001 | 0.0001 |
| **stage_new=IVa** | 0.82 | 0.79 | 2.28 (1.63-3.18) | 2.21 (1.66-2.94) | <0.0001 | <0.0001 |
| **stage_new=IVb** | 1.74 | 1.51 | 5.72 (3.07-10.65) | 4.52 (2.79-7.33) | <0.0001 | <0.0001 |
| **pack_years=5-25** | 0.18 | 0.27 | 1.2 (0.87-1.66) | 1.31 (1.01-1.7) | 0.2757 | 0.0459 |
| **pack_years=25-50** | 0.38 | 0.50 | 1.47 (1.1-1.95) | 1.65 (1.3-2.08) | 0.0085 | <0.0001 |
| **pack_years=>50** | 0.67 | 0.87 | 1.95 (1.45-2.64) | 2.38 (1.78-3.17) | <0.0001 | <0.0001 |
| **AGE=55-65** | 0.00 | 0.09 | 1 (0.76-1.33) | 1.09 (0.89-1.33) | 0.9815 | 0.4113 |
| **AGE=65-75** | 0.36 | 0.40 | 1.44 (1.08-1.92) | 1.49 (1.2-1.85) | 0.0143 | 0.0003 |
| **AGE=>75** | 0.62 | 0.75 | 1.87 (1.22-2.85) | 2.12 (1.56-2.89) | 0.0040 | <0.0001 |
|  |  |  |  |  |  |  |
|  |  |  |  |  |  |  |
| **OS model 2** |  |  |  |  |  |  |
|  | **β** | **β-pooled** | **HR (95%CI)** | **HR (95%CI)-pooled** | **p-value** | **p-value-pooled** |
| **Performance_score=1** | 0.54 | 0.45 | 1.72 (1.33-2.22) | 1.57 (1.27-1.94) | <0.0001 | <0.0001 |
| **Performance_score=>2** | 1.06 | 0.74 | 2.89 (2.12-3.94) | 2.1 (1.48-2.98) | <0.0001 | <0.0001 |
| **stage_new=II** | 0.40 | -0.04 | 1.49 (0.99-2.24) | 0.96 (0.63-1.48) | 0.0550 | 0.8697 |
| **stage_new=III** | 0.57 | 0.34 | 1.77 (1.19-2.64) | 1.4 (0.98-2.01) | 0.0051 | 0.0680 |
| **stage_new=IVa** | 0.68 | 0.47 | 1.97 (1.35-2.87) | 1.6 (1.16-2.21) | 0.0004 | 0.0049 |
| **stage_new=IVb** | 1.63 | 1.32 | 5.09 (2.69-9.62) | 3.76 (2.28-6.19) | <0.0001 | <0.0001 |
| **pack_years=5-25** | 0.17 | 0.27 | 1.19 (0.86-1.65) | 1.32 (1.02-1.7) | 0.2997 | 0.0354 |
| **pack_years=25-50** | 0.39 | 0.52 | 1.47 (1.1-1.97) | 1.68 (1.33-2.12) | 0.0091 | <0.0001 |
| **pack_years=>50** | 0.68 | 0.90 | 1.98 (1.46-2.67) | 2.45 (1.88-3.19) | <0.0001 | <0.0001 |
| **AGE=55-65** | 0.00 | 0.08 | 1 (0.76-1.33) | 1.08 (0.88-1.32) | 0.9752 | 0.4547 |
| **AGE=65-75** | 0.38 | 0.42 | 1.47 (1.1-1.96) | 1.53 (1.23-1.89) | 0.0098 | 0.0001 |
| **AGE=>75** | 0.65 | 0.80 | 1.92 (1.26-2.95) | 2.23 (1.64-3.04) | 0.0027 | <0.0001 |
| **T_stage=T3** | 0.18 | 0.39 | 1.2 (0.89-1.61) | 1.48 (1.09-2.01) | 0.2321 | 0.0143 |
| **T_stage=T4** | 0.25 | 0.53 | 1.28 (0.95-1.73) | 1.7 (1.27-2.29) | 0.1028 | 0.0005 |
|  |  |  |  |  |  |  |
|  |  |  |  |  |  |  |
| **OS model 3** |  |  |  |  |  |  |
|  | **β** | **β-pooled** | **HR (95%CI)** | **HR (95%CI)-pooled** | **p-value** | **p-value-pooled** |
| **Performance_score=1** | 0.54 | 0.44 | 1.71 (1.32-2.21) | 1.55 (1.25-1.92) | <0.0001 | <0.0001 |
| **Performance_score=>2** | 1.05 | 0.72 | 2.84 (2.09-3.88) | 2.06 (1.49-2.84) | <0.0001 | <0.0001 |
| **stage_new=II** | 0.35 | -0.10 | 1.42 (0.94-2.13) | 0.9 (0.59-1.37) | 0.0936 | 0.6261 |
| **stage_new=III** | 0.48 | 0.27 | 1.62 (1.07-2.44) | 1.31 (0.91-1.87) | 0.0212 | 0.1439 |
| **stage_new=IVa** | 0.44 | 0.28 | 1.55 (1.02-2.36) | 1.32 (0.95-1.83) | 0.0407 | 0.0994 |
| **stage_new=IVb** | 1.45 | 1.23 | 4.26 (2.22-8.18) | 3.41 (2.09-5.58) | <0.0001 | <0.0001 |
| **pack_years=5-25** | 0.17 | 0.27 | 1.18 (0.85-1.64) | 1.31 (1.01-1.69) | 0.3150 | 0.0400 |
| **pack_years=25-50** | 0.33 | 0.48 | 1.39 (1.03-1.86) | 1.61 (1.28-2.04) | 0.0297 | <0.0001 |
| **pack_years=>50** | 0.60 | 0.81 | 1.82 (1.34-2.47) | 2.25 (1.71-2.95) | 0.0001 | <0.0001 |
| **AGE=55-65** | 0.01 | 0.06 | 1.01 (0.76-1.34) | 1.07 (0.87-1.3) | 0.9468 | 0.5321 |
| **AGE=65-75** | 0.37 | 0.39 | 1.45 (1.08-1.94) | 1.48 (1.2-1.84) | 0.0135 | 0.0003 |
| **AGE=>75** | 0.61 | 0.74 | 1.84 (1.2-2.83) | 2.09 (1.53-2.86) | 0.0053 | <0.0001 |
| **T_stage=T3** | 0.15 | 0.39 | 1.17 (0.86-1.57) | 1.47 (1.1-1.99) | 0.3187 | 0.0116 |
| **T_stage=T4** | 0.28 | 0.57 | 1.32 (0.98-1.79) | 1.76 (1.33-2.34) | 0.0711 | 0.0001 |
| **site2=OPC-Naso-unknown** | -0.36 | -0.37 | 0.69 (0.53-0.91) | 0.69 (0.54-0.87) | 0.0076 | 0.0025 |
|  |  |  |  |  |  |  |
|  |  |  |  |  |  |  |
| **OS model 4** |  |  |  |  |  |  |
|  | **β** | **β-pooled** | **HR (95%CI)** | **HR (95%CI)-pooled** | **p-value** | **p-value-pooled** |
| **Performance_score=1** | 0.54 | 0.42 | 1.71 (1.32-2.22) | 1.52 (1.23-1.88) | <0.0001 | 0.0001 |
| **Performance_score=>2** | 1.04 | 0.70 | 2.84 (2.08-3.88) | 2.02 (1.48-2.77) | <0.0001 | <0.0001 |
| **stage_new=II** | 0.34 | -0.18 | 1.41 (0.92-2.14) | 0.84 (0.55-1.28) | 0.1115 | 0.4132 |
| **stage_new=III** | 0.49 | 0.17 | 1.63 (1.07-2.48) | 1.18 (0.83-1.68) | 0.0230 | 0.3611 |
| **stage_new=IVa** | 0.43 | 0.18 | 1.54 (0.99-2.39) | 1.19 (0.85-1.67) | 0.0550 | 0.3090 |
| **stage_new=IVb** | 1.58 | 0.59 | 4.87 (1.53-15.47) | 1.8 (0.83-3.93) | 0.0074 | 0.1395 |
| **pack_years=5-25** | 0.17 | 0.26 | 1.19 (0.86-1.64) | 1.3 (1.01-1.68) | 0.3075 | 0.0423 |
| **pack_years=25-50** | 0.33 | 0.47 | 1.39 (1.03-1.86) | 1.61 (1.27-2.02) | 0.0298 | <0.0001 |
| **pack_years=>50** | 0.60 | 0.83 | 1.82 (1.33-2.48) | 2.28 (1.74-3) | 0.0001 | <0.0001 |
| **AGE=55-65** | 0.01 | 0.07 | 1.01 (0.76-1.34) | 1.07 (0.88-1.31) | 0.9422 | 0.4987 |
| **AGE=65-75** | 0.37 | 0.41 | 1.45 (1.08-1.94) | 1.5 (1.21-1.86) | 0.0129 | 0.0002 |
| **AGE=>75** | 0.61 | 0.77 | 1.85 (1.2-2.84) | 2.16 (1.58-2.95) | 0.0052 | <0.0001 |
| **T_stage=T3** | 0.15 | 0.42 | 1.16 (0.86-1.57) | 1.52 (1.14-2.04) | 0.3274 | 0.0055 |
| **T_stage=T4** | 0.28 | 0.58 | 1.32 (0.97-1.78) | 1.79 (1.36-2.36) | 0.0736 | <0.0001 |
| **site2=OPC-Naso-unknown** | -0.37 | -0.42 | 0.69 (0.53-0.92) | 0.66 (0.52-0.83) | 0.0099 | 0.0005 |
| **N_stage=N2c** | 0.03 | 0.22 | 1.03 (0.76-1.39) | 1.25 (0.99-1.58) | 0.8457 | 0.0620 |
| **N_stage=N3** | -0.13 | 0.61 | 0.88 (0.35-2.21) | 1.84 (1.04-3.25) | 0.7791 | 0.0353 |

**eResults 1.2. Summary of variable selection and modelling considerations for Local Control (LC)**

Tumor site and HPV status were competing factors for LC prediction; HPV status and tumor site were highly correlated (Rho= 0.89; Chi-squared test: p-value < 2.2e-16; eTable 2 and C2). Because the univariable c-index performance was better for HPV status (c-index=0.62) than tumor site (c-index=0.61), tumor site was excluded from the analyses for LC.

In contrast to the OS prediction, the result from the variable selection for the LC Cox regression model was not consistent for every imputation subset: while T stage was selected in all iterations (eTable R1.2.2.), AJCC^8th^ stage was selected initially in many subset (eTable R1.2.1). Furthermore, performance score (7x), HPV status (9x) and pack years (5x) were selected. Since AJCC^8th^ stage depends on T stage and HPV status it is likely undesirable to have them in the same model. The forward variable selection on the complete data (eTable R1.2.3.), identified the T-stage, HPV status, Performance score as predictors for LC. While selected in some of the imputation sets (eTable R1.2.1.), pack years did not make it into the model in the smaller complete dataset, due to not meeting the likelihood-ratio test significance level (eTable R1.2.3. in grey letters).

The potential models that are tested are models with either T-stage or AJCC^8th^ stage combined with performance score and HPV status (eTable R1.2.4: LC model 1 and LC model 3). Additionally, the addition of pack years was tested (eTable R1.2.4: LC model 2 and LC model 4). The LC model 1 (with T stage, performance score, HPV, pack years) showed the best c-indices in the training set (c-index=0.74) and in external validation cohort 1 (c-index=0.71). The same model without pack years performed better in the second validation cohort (c-index=0.76), while LC model 4 with AJCC stage and pack years performed better on the independent test set. Nevertheless, the performance of LC model 1 performed best over all the cohorts.

The differences between the coefficients of the pooled models (obtained from the imputed data) versus those trained on complete training set were relatively large (eTable R1.2.5), which also translates to differences in patient stratification (Figure D2). This suggests that the coefficients may not be optimally estimated for LC prediction; this is likely due to the relatively low number of events (10.4%) and since 3 of the 4 variables needed imputation. For this model the coefficients estimated on the complete cases showed the best results.

In conclusion, the Cox regression model for LC with T-stage, performance score, HPV status, and pack years was selected as the final model.

**eTable R1.2.1. Forward selection in the different imputation** sets for likelihood ratio-test threshold of p<0.005*

*** *Bonferroni corrected p value= 0.05/10*

|  | **Variable 1** | **Variable 2** | **Variable 3** | **Variable 4** |
| --- | --- | --- | --- | --- |
| **Imputation set 1** | T_stage_LC | HPV.P16.status | Performance_score |  |
| **Imputation set 2** | stage_new | pack_years | T_stage_LC | HPV.P16.status |
| **Imputation set 3** | stage_new | Performance_score | T_stage_LC | HPV.P16.status |
| **Imputation set 4** | stage_new | pack_years | T_stage_LC | HPV.P16.status |
| **Imputation set 5** | T_stage_LC | HPV.P16.status | Performance_score |  |
| **Imputation set 6** | stage_new | Performance_score | T_stage_LC | pack_years |
| **Imputation set 7** | stage_new | Performance_score | T_stage_LC | HPV.P16.status |
| **Imputation set 8** | T_stage_LC | pack_years | HPV.P16.status | Performance_score |
| **Imputation set 9** | stage_new | T_stage_LC | HPV.P16.status | Performance_score |
| **Imputation set 10** | T_stage_LC | HPV.P16.status | pack_years |  |

**eTable R1.2.2 Frequency of selected variables**

|  | **frequency** |
| --- | --- |
| **Performance_score** | 7 |
| **T_stage_LC** | 10 |
| **HPV.P16.status** | 9 |
| **stage_new** | 6 |
| **pack_years** | 5 |
| **site2*** | 0 |
| **N_stage** | 0 |
| **AGE** | 0 |
| **Gender** | 0 |

*** excluded**

**eTable R1.2.3 Forward step-wise variable selection in complete cases** (n=1320) based on *likelihood ratio-test* for LC

|  |  |  |  |  |  |  |  |  |  |  |
| --- | --- | --- | --- | --- | --- | --- | --- | --- | --- | --- |
|  | **Local Control** | **(LC)** |  |  |  |  |  |  |  |  |
|  | ***variables*** | ***categories*** | ***coef*** | ***HR*** | ***p value*** | ***LLH*** | ***ratio-test*** | ***AIC*** | ***BIC*** | ***c-index*** |
| **step 1** | **T Stage** | T stageT2 | 1.54 | 4.65 | <0.0001 | -946 | <0.0001 | 1897 | 1906 | 0.66 |
|  |  | T stageT3 | 1.79 | 6.01 | <0.0001 |  |  |  |  |  |
|  |  | T stageT4 | 1.96 | 7.07 | <0.0001 |  |  |  |  |  |
| **step 2** | **T Stage** | T stageT2 | 1.48 | 4.38 | <0.0001 | -933 | <0.0001 | 1874 | 1886 | 0.72 |
|  |  | T stageT3 | 1.62 | 5.05 | <0.0001 |  |  |  |  |  |
|  |  | T stageT4 | 1.76 | 5.80 | <0.0001 |  |  |  |  |  |
|  | **HPV status** | HPV statusPositive | -0.87 | 0.42 | <0.0001 |  |  |  |  |  |
| **step 3** | **T Stage** | T stageT2 | 1.45 | 4.25 | <0.0001 | -926 | 0.0007 | 1864 | 1882 | 0.74 |
|  |  | T stageT3 | 1.51 | 4.54 | <0.0001 |  |  |  |  |  |
|  |  | T stageT4 | 1.61 | 5.00 | <0.0001 |  |  |  |  |  |
|  | **HPV status** | HPV statusPositive | -0.81 | 0.44 | <0.0001 |  |  |  |  |  |
|  | **Performance score** | Performance score1 | 0.47 | 1.60 | 0.0126 |  |  |  |  |  |
|  |  | Performance score>2 | 0.90 | 2.46 | 0.0002 |  |  |  |  |  |
| **step 4** | **T Stage** | T stageT2 | 1.44 | 4.20 | <0.0001 | -923 | 0.1430 | 1864 | 1891 | 0.74 |
|  |  | T stageT3 | 1.48 | 4.38 | <0.0001 |  |  |  |  |  |
|  |  | T stageT4 | 1.62 | 5.04 | <0.0001 |  |  |  |  |  |
|  | **HPV status** | HPV statusPositive | -0.69 | 0.50 | 0.0003 |  |  |  |  |  |
|  | **Performance score** | Performance score1 | 0.42 | 1.52 | 0.0276 |  |  |  |  |  |
|  |  | Performance score>2 | 0.80 | 2.23 | 0.0010 |  |  |  |  |  |
|  | **Pack years** | pack years5-25 | -0.04 | 0.96 | 0.8807 |  |  |  |  |  |
|  |  | pack years25-50 | 0.29 | 1.34 | 0.1858 |  |  |  |  |  |
|  |  | pack years>50 | 0.50 | 1.64 | 0.0403 |  |  |  |  |  |

**eTable R1.2.4 Performance (c-index) of potential LC models** in training and validation cohort

**Model variables:**

**LC Model 1:** Surv ~ T_stage + Performance_score + HPV_status + pack_years

**LC Model 2:** Surv ~ T_stage + Performance_score + HPV_status

**LC Model 3:** Surv ~ stage_new + Performance_score + HPV_status + pack_years

**LC Model 4:** Surv ~ stage_new + Performance_score + HPV_status

**LC Model 5:** Surv ~ stage_new + Performance_score + pack_years

N.b. coefficients in eTable R1.2.5.

|  |  | **Training** | **indep. Test** | **External val. 1** | **External val. 2** |
| --- | --- | --- | --- | --- | --- |
| **LC model 1** | **complete cases** | 0.74 (0.7-0.78) | 0.71 (0.58-0.84) | 0.70 (0.62-0.76) | 0.74 (0.59-0.89) |
|  | **imputed pooled** | 0.74 (0.66-0.82) | 0.72 (0.60-0.85) | 0.71 (0.64-0.78) | 0.73 (0.57-0.88) |
| **LC model 2** | **complete cases** | 0.74 (0.7-0.77) | 0.70 (0.57-0.84) | 0.68 (0.61-0.73) | 0.76 (0.61-0.9) |
|  | **imputed pooled** | 0.73 (0.66-0.8) | 0.71 (0.58-0.83) | 0.68 (0.62-0.74) | 0.76 (0.61-0.9) |
| **LC model 3** | **complete cases** | 0.71 (0.67-0.75) | 0.72 (0.61-0.84) | 0.64 (0.58-0.70) | 0.73 (0.57-0.88) |
|  | **imputed pooled** | 0.71 (0.63-0.79) | 0.73 (0.61-0.84) | 0.67 (0.61-0.73) | 0.71 (0.55-0.87) |
| **LC model 4** | **complete cases** | 0.72 (0.68-0.76) | 0.74 (0.62-0.85) | 0.68 (0.60-0.75) | 0.71 (0.56-0.86) |
|  | **imputed pooled** | 0.72 (0.63-0.8) | 0.74 (0.61-0.85) | 0.69 (0.61-0.77) | 0.69 (0.52-0.85) |
| **LC model 5** | **complete cases** | 0.72 (0.68-0.75) | 0.73 (0.60-0.85) | 0.69 (0.61-0.76) | 0.68 (0.52-0.83) |
|  | **imputed pooled** | 0.71 (0.63-0.79) | 0.73 (0.61-0.85) | 0.69 (0.62-0.76) | 0.67 (0.50-0.83) |

**eTable R1.2.5 Coefficients (β), Hazard ratio (HR) and p-values of potential LC models**

| **LC model 1** |  |  |  |  |  |  |
| --- | --- | --- | --- | --- | --- | --- |
|  | **β** | **β-pooled** | **HR (95%CI)** | **HR (95%CI)-pooled** | **p-value** | **p-value-pooled** |
| **T_stage_LC=T2** | 1.43 | 1.00 | 4.19 (2.19-8.03) | 2.73 (1.69-4.41) | <0.0001 | <0.0001 |
| **T_stage_LC=T3** | 1.47 | 1.17 | 4.36 (2.22-8.58) | 3.23 (1.93-5.4) | <0.0001 | <0.0001 |
| **T_stage_LC=T4** | 1.61 | 1.43 | 5.02 (2.56-9.83) | 4.16 (2.52-6.86) | <0.0001 | <0.0001 |
| **Performance_score=1** | 0.42 | 0.34 | 1.52 (1.05-2.22) | 1.4 (0.99-1.98) | 0.0276 | 0.0551 |
| **Performance_score=>2** | 0.80 | 0.53 | 2.23 (1.38-3.59) | 1.7 (1.07-2.7) | 0.0010 | 0.0262 |
| **HPV.P16.status=Positive** | -0.69 | -0.54 | 0.5 (0.34-0.73) | 0.58 (0.43-0.79) | 0.0003 | 0.0005 |
| **pack_years=5-25** | -0.04 | 0.24 | 0.96 (0.58-1.6) | 1.27 (0.86-1.86) | 0.8807 | 0.2251 |
| **pack_years=25-50** | 0.29 | 0.53 | 1.34 (0.87-2.08) | 1.7 (1.21-2.38) | 0.1858 | 0.0022 |
| **pack_years=>50** | 0.50 | 0.40 | 1.64 (1.02-2.64) | 1.49 (0.98-2.27) | 0.0403 | 0.0627 |
|  |  |  |  |  |  |  |
| **LC model 2** |  |  |  |  |  |  |
|  | **β** | **β-pooled** | **HR (95%CI)** | **HR (95%CI)-pooled** | **p-value** | **p-value-pooled** |
| **T_stage_LC=T2** | 1.47 | 1.01 | 4.37 (2.29-8.34) | 2.75 (1.7-4.43) | <0.0001 | <0.0001 |
| **T_stage_LC=T3** | 1.50 | 1.22 | 4.49 (2.29-8.8) | 3.37 (2.01-5.68) | <0.0001 | <0.0001 |
| **T_stage_LC=T4** | 1.59 | 1.43 | 4.91 (2.51-9.62) | 4.17 (2.52-6.9) | <0.0001 | <0.0001 |
| **Performance_score=1** | 0.44 | 0.39 | 1.55 (1.08-2.24) | 1.48 (1.05-2.09) | 0.0178 | 0.0248 |
| **Performance_score=>2** | 0.84 | 0.60 | 2.31 (1.45-3.68) | 1.83 (1.13-2.95) | 0.0004 | 0.0149 |
| **HPV.P16.status=Positive** | -0.81 | -0.62 | 0.45 (0.31-0.63) | 0.54 (0.4-0.72) | <0.0001 | <0.0001 |
|  |  |  |  |  |  |  |
| **LC model 3** |  |  |  |  |  |  |
|  | **β** | **β-pooled** | **HR (95%CI)** | **HR (95%CI)-pooled** | **p-value** | **p-value-pooled** |
| **stage_new=II** | 0.93 | 0.89 | 2.53 (1.38-4.63) | 2.44 (1.5-3.96) | 0.0027 | 0.0004 |
| **stage_new=III** | 0.90 | 0.92 | 2.45 (1.34-4.5) | 2.52 (1.55-4.09) | 0.0037 | 0.0002 |
| **stage_new=IVa** | 0.85 | 0.95 | 2.35 (1.27-4.35) | 2.58 (1.54-4.3) | 0.0065 | 0.0003 |
| **stage_new=IVb** | 1.49 | 1.32 | 4.46 (1.45-13.7) | 3.73 (1.44-9.67) | 0.0098 | 0.0067 |
| **Performance_score=1** | 0.49 | 0.42 | 1.63 (1.13-2.35) | 1.52 (1.09-2.11) | 0.0084 | 0.0141 |
| **Performance_score=>2** | 0.94 | 0.65 | 2.55 (1.61-4.03) | 1.91 (1.17-3.12) | <0.0001 | 0.0107 |
| **HPV.P16.status=Positive** | -0.66 | -0.34 | 0.52 (0.34-0.78) | 0.71 (0.5-1) | 0.0015 | 0.0487 |
|  |  |  |  |  |  |  |
| **LC model 4** |  |  |  |  |  |  |
|  | **β** | **β-pooled** | **HR (95%CI)** | **HR (95%CI)-pooled** | **p-value** | **p-value-pooled** |
| **stage_new=II** | 0.89 | 0.90 | 2.42 (1.31-4.49) | 2.45 (1.5-4) | 0.0049 | 0.0004 |
| **stage_new=III** | 0.88 | 0.89 | 2.41 (1.31-4.43) | 2.43 (1.5-3.94) | 0.0046 | 0.0003 |
| **stage_new=IVa** | 0.89 | 0.95 | 2.45 (1.32-4.55) | 2.59 (1.56-4.3) | 0.0047 | 0.0002 |
| **stage_new=IVb** | 1.49 | 1.31 | 4.46 (1.45-13.72) | 3.7 (1.43-9.59) | 0.0101 | 0.0071 |
| **Performance_score=1** | 0.46 | 0.36 | 1.58 (1.09-2.3) | 1.43 (1.03-2) | 0.0164 | 0.0351 |
| **Performance_score=>2** | 0.90 | 0.58 | 2.45 (1.53-3.92) | 1.78 (1.11-2.87) | 0.0002 | 0.0186 |
| **HPV.P16.status=Positive** | -0.51 | -0.24 | 0.6 (0.39-0.92) | 0.78 (0.55-1.12) | 0.0199 | 0.1776 |
| **pack_years=5-25** | -0.08 | 0.20 | 0.92 (0.56-1.53) | 1.22 (0.83-1.78) | 0.7548 | 0.3142 |
| **pack_years=25-50** | 0.33 | 0.55 | 1.39 (0.9-2.14) | 1.74 (1.24-2.43) | 0.1364 | 0.0012 |
| **pack_years=>50** | 0.49 | 0.40 | 1.63 (1.02-2.61) | 1.5 (0.99-2.26) | 0.0422 | 0.0548 |
|  |  |  |  |  |  |  |
| **LC model 5** |  |  |  |  |  |  |
|  | **β** | **β-pooled** | **HR (95%CI)** | **HR (95%CI)-pooled** | **p-value** | **p-value-pooled** |
| **stage_new=II** | 0.99 | 0.96 | 2.69 (1.47-4.95) | 2.6 (1.6-4.24) | 0.0014 | 0.0001 |
| **stage_new=III** | 1.06 | 0.97 | 2.9 (1.61-5.2) | 2.64 (1.66-4.2) | 0.0004 | <0.0001 |
| **stage_new=IVa** | 1.24 | 1.14 | 3.47 (2.01-6.01) | 3.12 (2.02-4.81) | <0.0001 | <0.0001 |
| **stage_new=IVb** | 1.79 | 1.48 | 5.98 (2-17.91) | 4.41 (1.75-11.13) | 0.0016 | 0.0017 |
| **Performance_score=1** | 0.45 | 0.36 | 1.56 (1.07-2.27) | 1.43 (1.03-1.98) | 0.0199 | 0.0331 |
| **Performance_score=>2** | 0.87 | 0.58 | 2.38 (1.49-3.81) | 1.78 (1.12-2.85) | 0.0003 | 0.0168 |
| **pack_years=5-25** | -0.06 | 0.21 | 0.94 (0.57-1.56) | 1.23 (0.84-1.8) | 0.8213 | 0.2907 |
| **pack_years=25-50** | 0.46 | 0.59 | 1.58 (1.04-2.4) | 1.8 (1.29-2.51) | 0.0320 | 0.0005 |
| **pack_years=>50** | 0.61 | 0.45 | 1.84 (1.16-2.92) | 1.57 (1.05-2.35) | 0.0096 | 0.0277 |

**eFigure R1.2 Patient stratification plots for local control risk.**

Low-risk classification was based on a 2-year local failure risk of <5% (green), intermediate risk <=5% and <20% (orange), and high risk >20%.

Local failure incidence seems to plateau differently per cohort, but overall this occurs 2-3 years after treatment. This is in line with clinical experience. In contrast to the other cohorts, for the MDACC cohorts first a plateau is visible, but it is followed by late tumor recurrences. The stratification as particularly well seen for the high-risk group, of whom the cumulative local failure rates varied between 25-50%. The number of patients selected as high-risk for local failure was variable per cohort, where the HPV negative rich UMCG cohort had proportionally many high-risk patients (19%) compared to the OPC only, high HPV positive PMH cohort (7%). Nevertheless, the LC model does select the high-risk patient well as ~50% of the patients in this group experience a local tumor failure. The low-risk group shows in all cohort high rates of local control, particularly for the first 6-8 years after treatment.

**
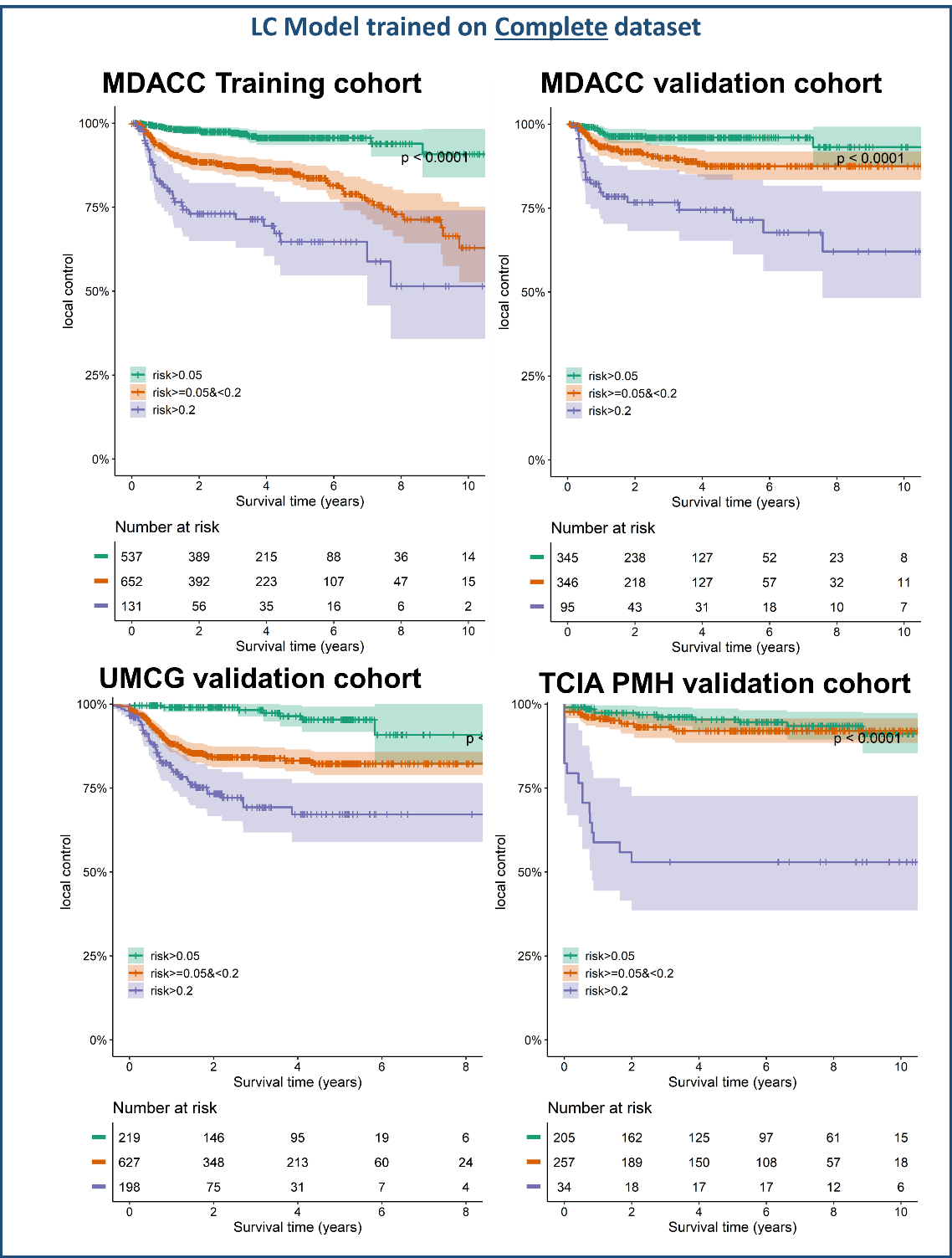
**

**eResults 1.3*.* Summary of variable selection and modelling considerations for Regional Control (RC)**

Similar to the prediction of LC, the RC variable results are not consistent. For the selected variables in the imputed dataset it seems that AJCC stage is the most frequently selected first variable (eTable R1.3.1), which is also the case for the forward selection results in the complete cases only (eTable R1.3.3). Performance score (6x) is the only variable that is additionally selected that is not a dependent variable for the AJCC stage calculation (eTable 2). Nonetheless, we tested the potential Cox RC models: 1) AJCC stage and performance score, plus 2) site, 3) HPV status 4) N stage, as well as 5) a model with N stage, performance score, and site (see eTable R1.3.4). The models with AJCC stage, performance and tumor site (RC model 2) or with N stage (RC model 4) seemed to work well. However, evaluating the stratification curves, it seemed that RC model 2 performed better to split the patients in 3 risk groups.

Also similar to the LC analyses, the pooled coefficients did not seem to benefit the stratification (eFigure R1.3), likely because they are excessively altered (eTable R1.3.5). This suggests that the coefficients are not optimally estimated. For this model a refit is advised on the full dataset, including the validation sets.

In conclusion the final RC model is based on the variables AJCC stage, performance, and tumor site.

**eTable R1.3.1 Forward selection in the different imputation** sets for likelihood ratio-test threshold of p<0.005*

*** *Bonferroni corrected p value= 0.05/10*

**w N stage**

|  | **Variable 1** | **Variable 2** | **Variable 3** | **Variable 4** | **Variable 5** |
| --- | --- | --- | --- | --- | --- |
| **Imputation set 1** | stage_new | N_stage | site |  |  |
| **Imputation set 2** | stage_new | N_stage | Performance_score | site | HPV.P16.status |
| **Imputation set 3** | N_stage | stage_new | site | HPV.P16.status | Performance_score |
| **Imputation set 4** | N_stage | stage_new | site | HPV.P16.status |  |
| **Imputation set 5** | stage_new | N_stage | site |  |  |
| **Imputation set 6** | stage_new | N_stage | site | HPV.P16.status | Performance_score |
| **Imputation set 7** | stage_new | N_stage | Performance_score | site |  |
| **Imputation set 8** | stage_new | N_stage | site | HPV.P16.status | Performance_score |
| **Imputation set 9** | stage_new | N_stage | site | HPV.P16.status |  |
| **Imputation set 10** | stage_new | N_stage | site | Performance_score |  |

**eTable R1.3.2 Frequency of selected variables**

|  | **Frequency** |
| --- | --- |
| **Performance_score** | 6 |
| **T_stage_LC** | 0 |
| **HPV.P16.status** | 6 |
| **stage_new** | 10 |
| **pack_years** | 0 |
| **site** | 10 |
| **N_stage** | 10 |
| **AGE** | 0 |
| **Gender** | 0 |

**eTable R1.3.3 Forward step-wise variable selection in complete cases** (n=1320) based on *likelihood ratio-test* for RC

|  | **Regional Control** | **(RC)** |  |  |  |  |  |  |  |  |
| --- | --- | --- | --- | --- | --- | --- | --- | --- | --- | --- |
|  | ***variables*** | ***categories*** | ***coef*** | ***HR*** | ***p value*** | ***LLH*** | ***ratio-test*** | ***AIC*** | ***BIC*** | ***c-index*** |
| **step 1** | **AJCC stage** | stage newII | 0.66 | 1.94 | 0.0973 | -752 | <0.0001 | 1511 | 1522 | 0.70 |
|  |  | stage newIII | 1.16 | 3.19 | 0.0012 |  |  |  |  |  |
|  |  | stage newIVa | 1.84 | 6.27 | <0.0001 |  |  |  |  |  |
|  |  | stage newIVb | 2.83 | 17.00 | <0.0001 |  |  |  |  |  |
| **step 2** | **AJCC stage** | stage newII | 0.61 | 1.83 | 0.1335 | -740 | 0.0004 | 1499 | 1523 | 0.72 |
|  |  | stage newIII | 1.06 | 2.90 | 0.0048 |  |  |  |  |  |
|  |  | stage newIVa | 1.67 | 5.34 | <0.0001 |  |  |  |  |  |
|  |  | stage newIVb | 2.70 | 14.86 | <0.0001 |  |  |  |  |  |
|  | **site** | siteLarynx | -0.10 | 0.90 | 0.7688 |  |  |  |  |  |
|  |  | siteNasopharynx | -16.42 | 0.00 | 0.9929 |  |  |  |  |  |
|  |  | siteOPC | -0.79 | 0.46 | 0.0392 |  |  |  |  |  |
|  |  | siteOral Cavity | -0.98 | 0.38 | 0.0076 |  |  |  |  |  |
|  |  | siteUnkown primary | -1.37 | 0.25 | 0.0814 |  |  |  |  |  |
| **step 3** | **AJCC stage** | stage newII | 0.54 | 1.71 | 0.1860 | -735 | 0.0037 | 1491 | 1521 | 0.74 |
|  |  | stage newIII | 0.95 | 2.59 | 0.0115 |  |  |  |  |  |
|  |  | stage newIVa | 1.53 | 4.61 | <0.0001 |  |  |  |  |  |
|  |  | stage newIVb | 2.52 | 12.41 | <0.0001 |  |  |  |  |  |
|  | **site** | siteLarynx | -0.03 | 0.97 | 0.9406 |  |  |  |  |  |
|  |  | siteNasopharynx | -16.40 | 0.00 | 0.9943 |  |  |  |  |  |
|  |  | siteOPC | -0.68 | 0.51 | 0.0769 |  |  |  |  |  |
|  |  | siteOral Cavity | -0.86 | 0.42 | 0.0199 |  |  |  |  |  |
|  |  | siteUnkown primary | -1.13 | 0.32 | 0.1522 |  |  |  |  |  |
|  | **Performance score** | Performance score1 | 0.59 | 1.80 | 0.0077 |  |  |  |  |  |
|  |  | Performance score>2 | 0.85 | 2.33 | 0.0027 |  |  |  |  |  |

**eTable R1.3.4 Performance (c-index) of potential RC models** in training and validation cohort

**Model variables:**

**RC Model 1:** Surv(RC) ~ stage_new + Performance_score

**RC Model 2:** Surv(RC) ~ stage_new + Performance_score + site

**RC Model 3:** Surv(RC) ~ stage_new + Performance_score + HPV_status

**RC Model 5:** Surv(RC) ~ stage_new + Performance_score + N_stage

**RC Model 4:** Surv(RC) ~ N_stage + Performance_score + site

N.b. coefficients in eTable R1.3.5.

|  |  | **Training** | **indep. Test** | **External val. 1** | **External val. 2** |
| --- | --- | --- | --- | --- | --- |
| **RC model 1** | **complete cases** | 0.73 (0.68-0.77) | 0.72 (0.56-0.87) | 0.69 (0.61-0.76) | 0.71 (0.48-0.94) |
|  | **imputed pooled** | 0.73 (0.64-0.82) | 0.72 (0.56-0.87) | 0.68 (0.61-0.76) | 0.72 (0.49-0.94) |
| **RC model 2** | **complete cases** | 0.74 (0.69-0.78) | 0.73 (0.57-0.89) | 0.7 (0.62-0.77) | 0.71 (0.48-0.94) |
|  | **imputed pooled** | 0.74 (0.64-0.83) | 0.72 (0.55-0.88) | 0.7 (0.62-0.78) | 0.71 (0.47-0.94) |
| **RC model 3** | **complete cases** | 0.73 (0.69-0.78) | 0.71 (0.55-0.86) | 0.69 (0.57-0.81) | 0.71 (0.49-0.94) |
|  | **imputed pooled** | 0.73 (0.63-0.82) | 0.71 (0.55-0.86) | 0.69 (0.56-0.81) | 0.69 (0.44-0.92) |
| **RC model 4** | **complete cases** | 0.73 (0.69-0.77) | 0.71 (0.56-0.86) | 0.71 (0.63-0.78) | 0.73 (0.51-0.94) |
|  | **imputed pooled** | 0.72 (0.63-0.81) | 0.69 (0.54-0.83) | 0.71 (0.63-0.78) | 0.71 (0.48-0.94) |
| **RC model 5** | **complete cases** | 0.72 (0.68-0.77) | 0.71 (0.55-0.86) | 0.62 (0.53-0.71) | 0.67 (0.43-0.91) |
|  | **imputed pooled** | 0.73 (0.64-0.81) | 0.69 (0.54-0.84) | 0.64 (0.55-0.73) | 0.67 (0.43-0.91) |

**eTable R1.3.5. Coefficients (β), Hazard ratio (HR) and p-values of potential RC models**

| **RC model 1** |  |  |  |  |  |  |  |  |
| --- | --- | --- | --- | --- | --- | --- | --- | --- |
|  | **β** | **β-pooled** | **HR (95%CI)** | **HR (95%CI)-pooled** | **p-value** | **p-value-pooled** | **p-value** | **p-value-pooled** |
| **stage_new=II** | 0.51 | 0.65 | 1.66 (0.76-3.64) | 1.91 (1.1-3.33) | 0.21 | 0.02 | 0.2073 | 0.0226 |
| **stage_new=III** | 1.03 | 0.86 | 2.79 (1.38-5.66) | 2.37 (1.38-4.05) | 0.00 | 0.00 | 0.0044 | 0.0017 |
| **stage_new=IVa** | 1.66 | 1.34 | 5.24 (2.8-9.81) | 3.82 (2.37-6.13) | 0.00 | 0.00 | <0.0001 | <0.0001 |
| **stage_new=IVb** | 2.62 | 2.36 | 13.7 (5.34-35.17) | 10.55 (4.94-22.55) | 0.00 | 0.00 | <0.0001 | <0.0001 |
| **Performance_score=1** | 0.69 | 0.60 | 1.99 (1.29-3.05) | 1.81 (1.22-2.69) | 0.00 | 0.00 | 0.0017 | 0.0032 |
| **Performance_score=>2** | 0.92 | 0.66 | 2.52 (1.46-4.35) | 1.93 (1.17-3.17) | 0.00 | 0.01 | 0.0010 | 0.0100 |
| **RC model 2** |  |  |  |  |  |  |  |  |
|  | **β** | **β-pooled** | **HR (95%CI)** | **HR (95%CI)-pooled** | **p-value** | **p-value-pooled** | **p-value** | **p-value-pooled** |
| **stage_new=II** | 0.44 | 0.63 | 1.56 (0.7-3.46) | 1.88 (1.07-3.29) | 0.28 | 0.03 | 0.2774 | 0.0271 |
| **stage_new=III** | 0.98 | 0.96 | 2.68 (1.28-5.59) | 2.61 (1.5-4.54) | 0.01 | 0.00 | 0.0089 | 0.0007 |
| **stage_new=IVa** | 1.57 | 1.40 | 4.79 (2.34-9.81) | 4.08 (2.33-7.11) | 0.00 | 0.00 | <0.0001 | <0.0001 |
| **stage_new=IVb** | 2.57 | 2.47 | 13 (4.76-35.55) | 11.79 (5.21-26.69) | 0.00 | 0.00 | <0.0001 | <0.0001 |
| **Performance_score=1** | 0.57 | 0.53 | 1.77 (1.15-2.73) | 1.7 (1.14-2.54) | 0.01 | 0.01 | 0.0093 | 0.0092 |
| **Performance_score=>2** | 0.79 | 0.60 | 2.21 (1.27-3.84) | 1.83 (1.11-3) | 0.00 | 0.02 | 0.0049 | 0.0175 |
| **site=Larynx** | -0.12 | -0.31 | 0.89 (0.45-1.75) | 580.74 (0.37-1.46) | 0.73 | 0.38 | 0.7343 | 0.3780 |
| **site=Nasopharynx** | -5.00 | -16.03 | 0.01 (0-21498.48) | 0 (0-Inf) | 0.99 | 0.99 | 0.9932 | 0.9927 |
| **site=OPC** | -0.65 | -0.49 | 0.52 (0.25-1.11) | 0.61 (0.3-1.24) | 0.09 | 0.17 | 0.0898 | 0.1737 |
| **site=Oral Cavity** | -0.85 | -0.81 | 0.43 (0.21-0.88) | 0.44 (0.22-0.9) | 0.02 | 0.02 | 0.0203 | 0.0240 |
| **site=Unkown_primary** | -1.14 | -1.80 | 0.32 (0.07-1.51) | 0.17 (0.04-0.77) | 0.15 | 0.02 | 0.1493 | 0.0223 |
| **RC model 3** |  |  |  |  |  |  |  |  |
|  | **β** | **β-pooled** | **HR (95%CI)** | **HR (95%CI)-pooled** | **p-value** | **p-value-pooled** | **p-value** | **p-value-pooled** |
| **stage_new=II** | 0.57 | 0.78 | 1.76 (0.8-3.89) | 2.17 (1.23-3.83) | 0.16 | 0.01 | 0.1598 | 0.0075 |
| **stage_new=III** | 1.12 | 1.04 | 3.07 (1.49-6.31) | 2.82 (1.63-4.86) | 0.00 | 0.00 | 0.0023 | 0.0002 |
| **stage_new=IVa** | 1.86 | 1.80 | 6.41 (3.17-12.98) | 6.05 (3.49-10.47) | 0.00 | 0.00 | <0.0001 | <0.0001 |
| **stage_new=IVb** | 2.78 | 2.78 | 16.2 (6.09-43.1) | 16.1 (7.32-35.42) | 0.00 | 0.00 | <0.0001 | <0.0001 |
| **Performance_score=1** | 0.70 | 0.61 | 2.01 (1.31-3.09) | 1.83 (1.24-2.7) | 0.00 | 0.00 | 0.0014 | 0.0024 |
| **Performance_score=>2** | 0.94 | 0.69 | 2.56 (1.48-4.43) | 1.99 (1.23-3.24) | 0.00 | 0.01 | 0.0008 | 0.0056 |
| **HPV.P16.status=Positive** | 0.28 | 0.59 | 1.32 (0.84-2.07) | 1.81 (1.21-2.71) | 0.22 | 0.00 | 0.2228 | 0.0042 |
| **RC model 4** |  |  |  |  |  |  |  |  |
|  | **β** | **β-pooled** | **HR (95%CI)** | **HR (95%CI)-pooled** | **p-value** | **p-value-pooled** | **p-value** | **p-value-pooled** |
| **stage_new=II** | 0.40 | 0.40 | 1.49 (0.66-3.32) | 1.49 (0.85-2.62) | 0.34 | 0.16 | 0.3355 | 0.1601 |
| **stage_new=III** | 0.88 | 0.53 | 2.41 (1.15-5.05) | 1.69 (0.96-2.98) | 0.02 | 0.07 | 0.0193 | 0.0691 |
| **stage_new=IVa** | 1.56 | 1.12 | 4.77 (2.5-9.09) | 3.08 (1.88-5.04) | 0.00 | 0.00 | <0.0001 | <0.0001 |
| **stage_new=IVb** | 1.86 | 0.95 | 6.4 (1.52-26.88) | 2.6 (0.83-8.08) | 0.01 | 0.10 | 0.0113 | 0.1000 |
| **Performance_score=1** | 0.67 | 0.56 | 1.95 (1.27-2.99) | 1.75 (1.18-2.59) | 0.00 | 0.01 | 0.0024 | 0.0057 |
| **Performance_score=>2** | 0.89 | 0.62 | 2.43 (1.4-4.2) | 1.86 (1.16-3) | 0.00 | 0.01 | 0.0016 | 0.0107 |
| **N_stage=N2c** | 0.31 | 0.68 | 1.37 (0.88-2.13) | 1.98 (1.37-2.85) | 0.17 | 0.00 | 0.1659 | 0.0002 |
| **N_stage=N3** | 0.77 | 1.41 | 2.16 (0.73-6.41) | 4.1 (1.86-9.06) | 0.17 | 0.00 | 0.1652 | 0.0005 |
| **RC model 5** |  |  |  |  |  |  |  |  |
|  | **β** | **β-pooled** | **HR (95%CI)** | **HR (95%CI)-pooled** | **p-value** | **p-value-pooled** | **p-value** | **p-value-pooled** |
| **N_stage=N2c** | 0.89 | 0.99 | 2.43 (1.67-3.53) | 2.7 (1.92-3.79) | 0.00 | 0.00 | <0.0001 | <0.0001 |
| **N_stage=N3** | 1.72 | 1.71 | 5.6 (3.29-9.52) | 5.53 (3.42-8.93) | 0.00 | 0.00 | <0.0001 | <0.0001 |
| **Performance_score=1** | 0.58 | 0.53 | 1.78 (1.22-2.61) | 1.69 (1.13-2.54) | 0.00 | 0.01 | 0.0029 | 0.0114 |
| **Performance_score=>2** | 0.84 | 0.61 | 2.31 (1.41-3.81) | 1.84 (1.15-2.94) | 0.00 | 0.01 | 0.0010 | 0.0113 |
| **site=Larynx** | -0.39 | -0.53 | 0.68 (0.35-1.33) | 0.59 (0.3-1.16) | 0.26 | 0.12 | 0.2564 | 0.1245 |
| **site=Nasopharynx** | -5.47 | -16.20 | 0 (0-83298.67) | 0 (0-Inf) | 0.99 | 0.99 | 0.9937 | 0.9928 |
| **site=OPC** | -1.40 | -1.21 | 0.25 (0.13-0.47) | 0.3 (0.16-0.56) | 0.00 | 0.00 | <0.0001 | 0.0002 |
| **site=Oral Cavity** | -0.89 | -0.83 | 0.41 (0.2-0.84) | 0.44 (0.22-0.88) | 0.02 | 0.02 | 0.0150 | 0.0213 |
| **site=Unkown_primary** | -1.44 | -1.98 | 0.24 (0.05-1.07) | 0.14 (0.03-0.64) | 0.06 | 0.01 | 0.0620 | 0.0110 |

**eFigure R1.3. Patient stratification plots for regional control risk**

Low risk classification was based on a 2-year regional tumor failure risk of <5% (green), intermediate risk <=5% and <20% (orange), and high risk >20%.

Similar to local control, regional control rates plateau typically at 2-3 year after treatment. The models do well to stratify the low-risk patients that do not very low incidences of regional failure. Also, the high-risk patients show 5 year regional failure incidences between 25%-50%.

**
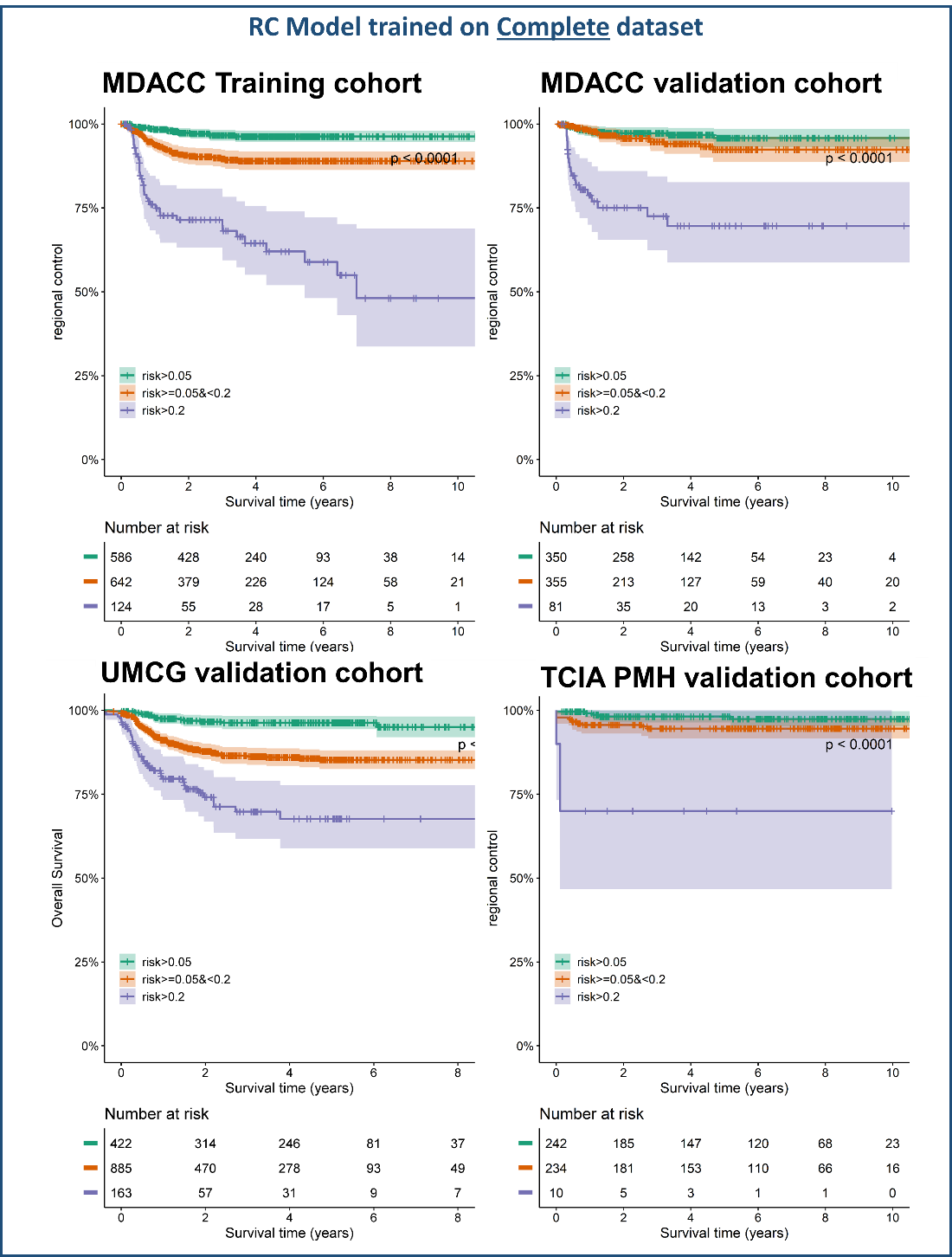
**

**eFigure 2. Calibration plot for the clinical prediction models for the different cohorts**

Per design, the calibration of all the clinical models in the training cohort is good for 2 years risk: both the calibration line (dotted blue line) and the data points lay on the diagonal line, meaning that the predicted rates correspond to the actual death, local or regional failure rates. This is also seen in non-significance of the Hosmer-Lemeshow test (i.e., p>0.05). Overall, the models show adequate calibration in the independent test and external cohorts. Significant deviations in the predicted and accrual rates were seen for OS model in the external validation cohorts, where the observed rates were systematically higher than the predicted mortality rates. Nevertheless, the calibration data points are fitted well on the calibration line, meaning that with a multiplication correction the prediction can be matched well. Additionally, the RC also showed some deviation from the diagonal line in the independent test cohort, but by visual investigating of the calibration plot this the impact seems minimal.

**
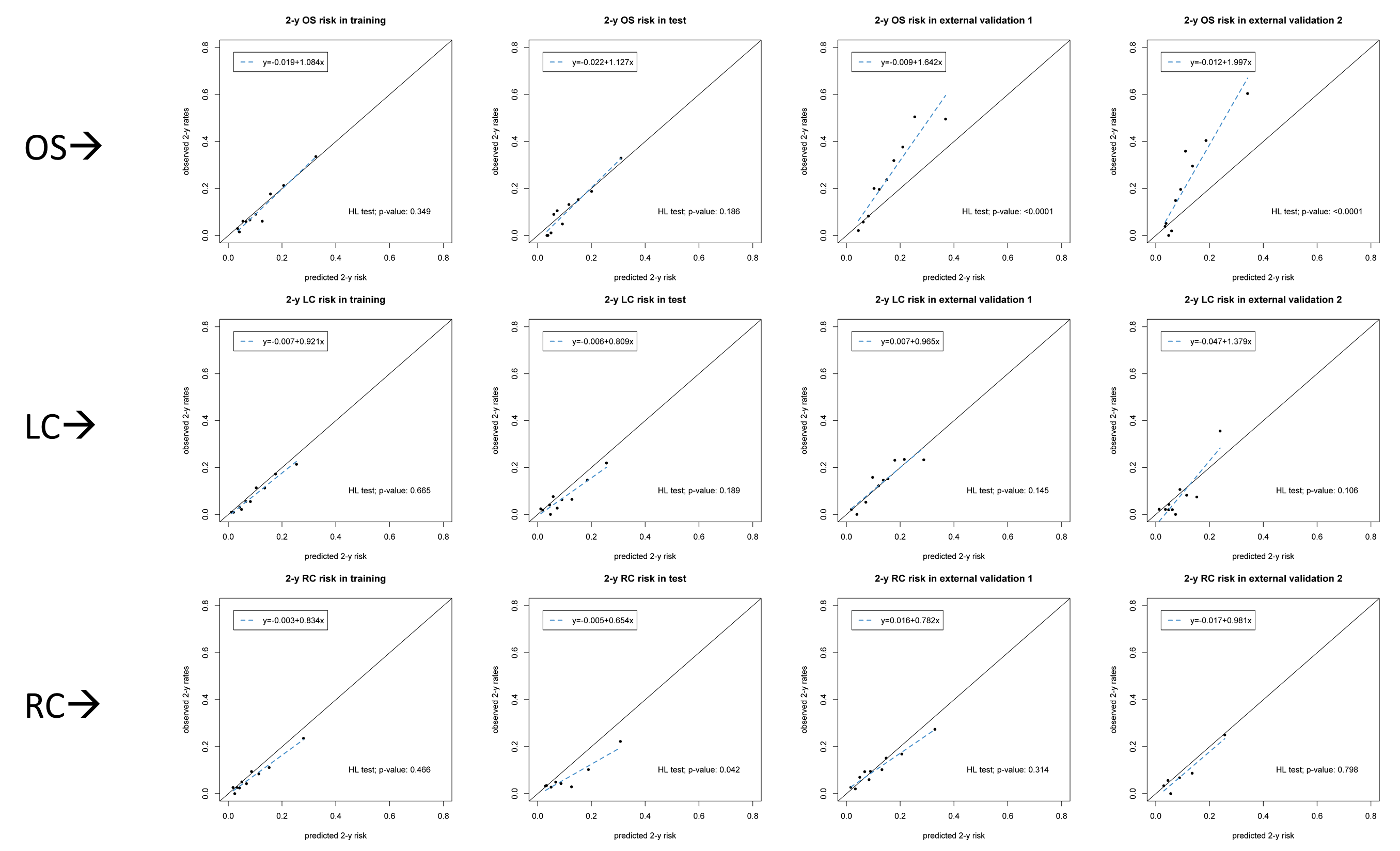
**

**eFigure 3. Calibration plot of the clinical prediction models for the different tumor site**

**
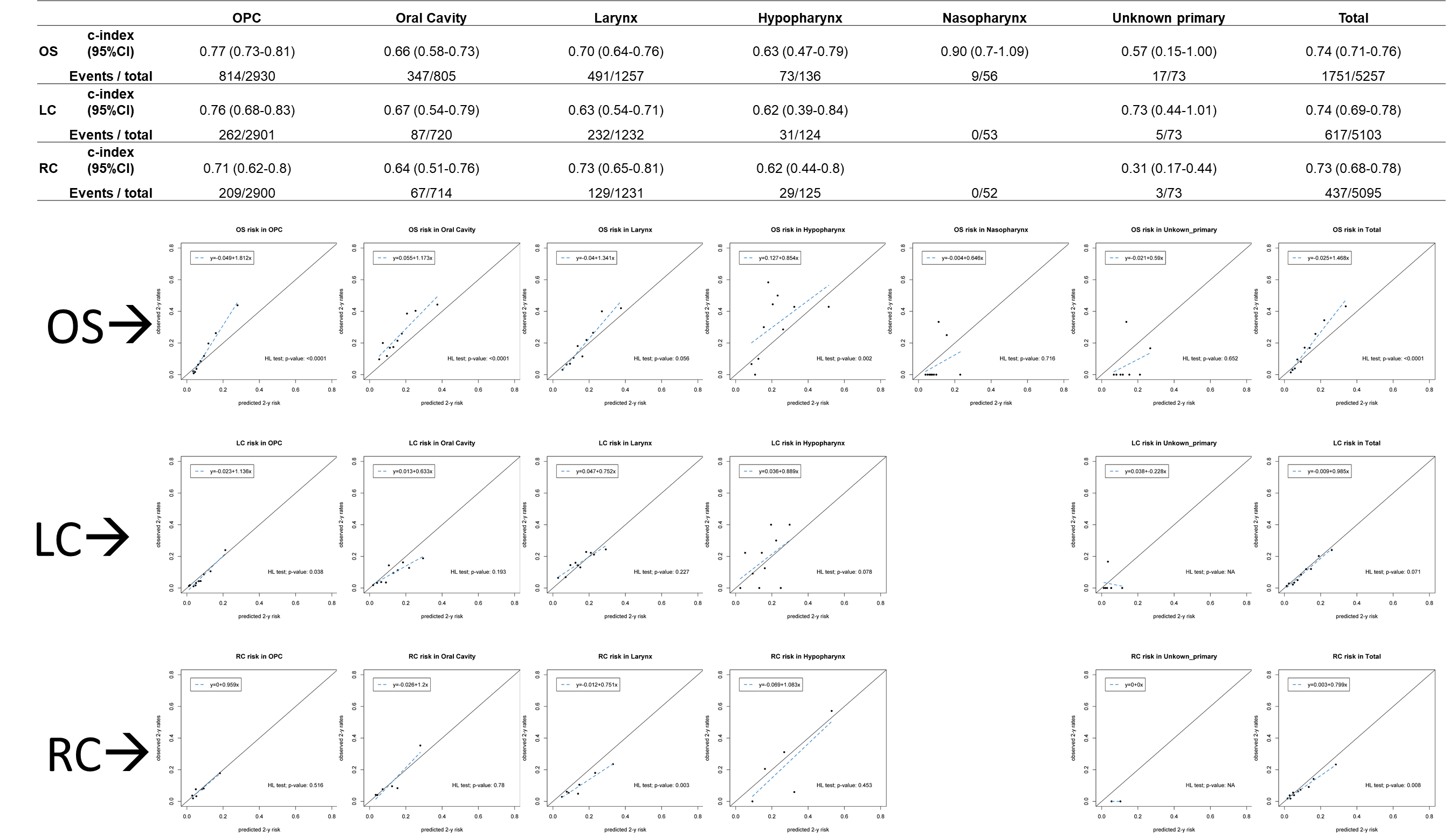
**

**eResults 2. Image biomarker selection**

**eResults 2.1. Summary of variable selection and modelling considerations for Geometric features**

For the geometric radiomics feature selection, 455 patients were used for training (no restrictions on metal artifacts were applied), and 229 and 430 patients for external validation cohort 1 and 2, respectively.

*The univariable analyses (****eTable R2.1.1****)* in the training cohort show a significant relationship between the vast majority of geometric image biomarkers of the primary tumor and the endpoints OS (17 out of 19 features) and LC (16 out of 19 features). For RC, less geometric image biomarkers are significant, namely 9 out of 20. The minor axis length was the most significant factor for OS and LC in the univariable analyses (p<0.0001).

*The forward step-wise variable selection (****eTable R2.1.2****)* in the training cohort (455 MDACC patients) identified the clinical risk (i.e., linear predictor (LP) of the clinical model) as first for all endpoints. For OS, ‘Minor_axis_length’ significantly added to the LP (p=0.004). No addition feature was selected. For LC, ‘Minor_axis_length’ also significantly added to the LP (p=0.04). No geometric feature significantly added to the LP of the RC model.

*Bootstrapped step-wise forward variable selection for the Geometric features* (***eFigure R2.1.1)*** in the training cohort (455 MDACC patients) selected the ‘minor axis length’ of the primary tumor as the most frequently (727 out of 1000 samples) selected as first geometric predictor for OS. This was also the case for LC, yet the frequency was lower (415 out of 1000 samples). Overall, the frequency of selected geometric features was low for RC, with the Volume density as most frequently selected (184 times).

*The performance of potential clinical+geometric feature models* (**eTable R2.1.3**) was compared to the clinical model in the training, and two validation cohort. For OS, the c-index improved from c-index=0.72 [0.63-0.81] with the clinical model to c-index=0.73 [0.64-0.81]. While the validation c-index increase was more pronounce in the UMCG cohort (from 0.71 (0.62-0.81) to 0.74 (0.64-0.83)), no increase in performance was seen in the MGH validation cohort (from 0.74 [0.67-0.80] to 0.74 [0.67-0.81]). For LC, the clinical+geometric model did not show improvement in c-index in the validation cohorts, thus did not validate well. Also, no improvement was seen with ‘Volume density’ in the validation cohort for RC, in contrast to 'Spherical_Disproportion'. However, the latter was not a significantly addition to LP in the training cohort (likelihood ratio test; p=0.14).

**eFigure R2.1.1 Frequency plot of image variables**. Variables were selected in 1000 bootstrap sample of the training cohort
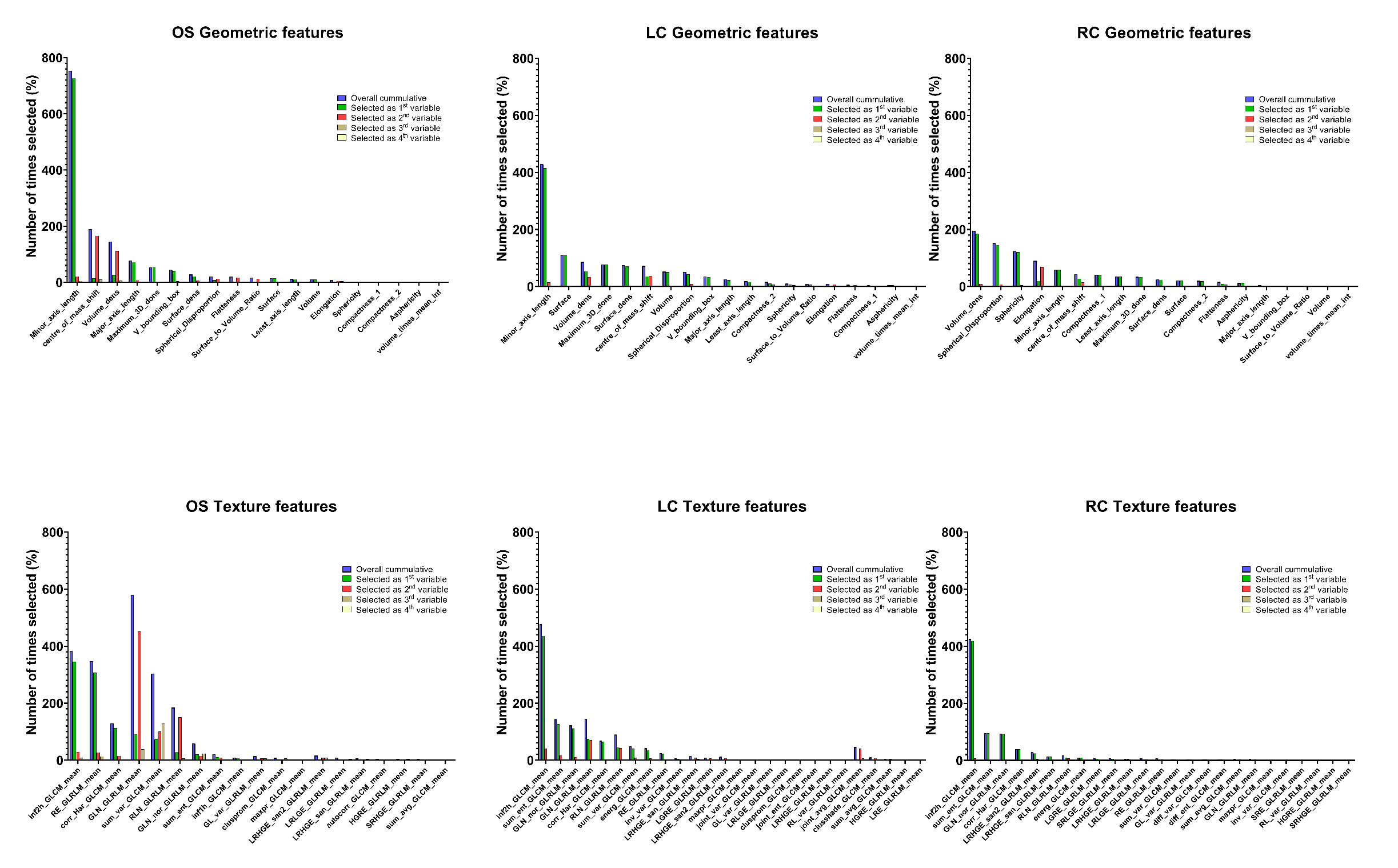


**eTable R2.1.1 Univariable analyses of geometric features**

| Overall survival | β | HR (95% CI) | p-value |  | Local control | β | HR (95% CI) | p-value |  | Regional Control | β | HR (95% CI) | p-value |
| --- | --- | --- | --- | --- | --- | --- | --- | --- | --- | --- | --- | --- | --- |
| **OS lp** | 0.99 | 2.68 (2.15-3.35) | **<0.0001** |  | **LC lp** | 1.00 | 2.72 (1.86-3.99) | **<0.0001** |  | **RC lp** | 0.73 | 2.08 (1.5-2.88) | **<0.0001** |
| **Minor axis length** | 0.50 | 1.65 (1.43-1.9) | **<0.0001** |  | **Minor axis length** | 0.53 | 1.7 (1.36-2.11) | **<0.0001** |  | **Sphericity** | -0.40 | 0.67 (0.5-0.9) | **0.008** |
| **V bounding box** | 0.44 | 1.55 (1.37-1.76) | **<0.0001** |  | **Surface** | 0.47 | 1.6 (1.3-1.97) | **0.0001** |  | **Spherical Disproportion** | 0.33 | 1.4 (1.12-1.75) | **0.009** |
| **Volume** | 0.40 | 1.49 (1.33-1.66) | **<0.0001** |  | **V bounding box** | 0.45 | 1.57 (1.3-1.9) | **0.0003** |  | **Asphericity** | 0.33 | 1.4 (1.12-1.75) | **0.009** |
| **volume times mean int** | 0.40 | 1.49 (1.33-1.67) | **<0.0001** |  | **Maximum 3D done** | 0.47 | 1.6 (1.26-2.05) | **0.0004** |  | **Compactness 1** | -0.40 | 0.67 (0.5-0.91) | **0.01** |
| **Surface** | 0.40 | 1.49 (1.31-1.69) | **<0.0001** |  | **Volume** | 0.40 | 1.5 (1.26-1.78) | **0.0007** |  | **Volume dens** | -0.38 | 0.68 (0.49-0.95) | **0.019** |
| **Maximum 3D done** | 0.44 | 1.56 (1.33-1.82) | **<0.0001** |  | **volume times mean int** | 0.40 | 1.5 (1.26-1.78) | **0.0007** |  | **Compactness 2** | -0.37 | 0.69 (0.5-0.96) | **0.023** |
| **Major axis length** | 0.41 | 1.51 (1.31-1.73) | **<0.0001** |  | **Least axis length** | 0.42 | 1.53 (1.23-1.88) | **0.001** |  | **Minor axis length** | 0.32 | 1.38 (1.06-1.8) | **0.032** |
| **Least axis length** | 0.41 | 1.51 (1.31-1.73) | **<0.0001** |  | **Major axis length** | 0.37 | 1.44 (1.19-1.76) | **0.002** |  | **Maximum 3D done** | 0.31 | 1.37 (1.04-1.8) | **0.036** |
| **Surface dens** | -0.46 | 0.63 (0.5-0.79) | **<0.0001** |  | **Surface dens** | -0.49 | 0.61 (0.44-0.86) | **0.002** |  | **Surface** | 0.28 | 1.32 (1.04-1.68) | **0.041** |
| **Asphericity** | 0.29 | 1.33 (1.15-1.54) | **0.0004** |  | **Spherical Disproportion** | 0.34 | 1.4 (1.11-1.77) | **0.008** |  | Least axis length | 0.28 | 1.32 (1.01-1.71) | 0.069 |
| **Spherical Disproportion** | 0.29 | 1.33 (1.15-1.54) | **0.0004** |  | **Asphericity** | 0.34 | 1.4 (1.11-1.77) | **0.008** |  | V bounding box | 0.27 | 1.32 (1.01-1.71) | 0.079 |
| **Volume dens** | -0.31 | 0.73 (0.6-0.89) | **0.001** |  | **Sphericity** | -0.34 | 0.71 (0.55-0.92) | **0.012** |  | Surface dens | -0.28 | 0.75 (0.53-1.07) | 0.097 |
| **Sphericity** | -0.29 | 0.75 (0.63-0.89) | **0.001** |  | **Compactness 1** | -0.34 | 0.71 (0.54-0.93) | **0.013** |  | Major axis length | 0.22 | 1.25 (0.95-1.64) | 0.144 |
| **Compactness 1** | -0.28 | 0.76 (0.64-0.9) | **0.002** |  | **Compactness 2** | -0.33 | 0.72 (0.54-0.96) | **0.02** |  | Volume | 0.24 | 1.27 (0.97-1.67) | 0.146 |
| **centre of mass shift** | 0.22 | 1.25 (1.12-1.39) | **0.003** |  | **Volume dens** | -0.33 | 0.72 (0.54-0.96) | **0.023** |  | volume times mean int | 0.24 | 1.27 (0.97-1.67) | 0.147 |
| **Compactness 2** | -0.25 | 0.78 (0.65-0.93) | **0.006** |  | **Surface to Volume Ratio** | -0.32 | 0.73 (0.53-1) | **0.038** |  | centre of mass shift | 0.15 | 1.16 (0.95-1.42) | 0.223 |
| **Surface to Volume Ratio** | -0.27 | 0.76 (0.62-0.94) | **0.008** |  | centre of mass shift | 0.18 | 1.2 (1.03-1.4) | 0.061 |  | Elongation | 0.16 | 1.17 (0.87-1.58) | 0.292 |
| Elongation | 0.10 | 1.11 (0.93-1.32) | 0.246 |  | Elongation | 0.11 | 1.12 (0.86-1.46) | 0.4 |  | Surface to Volume Ratio | -0.08 | 0.92 (0.67-1.27) | 0.605 |
| Flatteness | -0.01 | 0.99 (0.83-1.18) | 0.938 |  | Flatteness | 0.06 | 1.06 (0.82-1.37) | 0.656 |  | Flatteness | 0.07 | 1.07 (0.8-1.43) | 0.652 |

**eTable R2.1**.**2** Forward step-wise variable selection geometric

| ***Endpoint*** | ***modelling*** | ***variables*** | ***coef*** | ***HR*** | ***p value*** | ***LLH*** | ***ratio-test*** | ***AIC*** | ***BIC*** | ***c-index*** |
| --- | --- | --- | --- | --- | --- | --- | --- | --- | --- | --- |
| Overall Survival (OS) | step_1 | clinical risk (LP) | 0.99 | 2.68 | <0.0001 | -704.80 | **<0.0001** | 1411.60 | 1414.48 | 0.72 |
|  | step_2 | clinical risk (LP) + | 0.87 | 2.40 | <0.0001 | -700.66 | **0.0040** | 1405.32 | 1411.08 | 0.73 |
|  |  | Minor_axis_length | 0.25 | 1.29 | 0.0021 |  |  |  |  |  |
|  | step_3 | clinical risk (LP) + | 0.91 | 2.49 | <0.0001 | -699.34 | 0.1037 | 1404.68 | 1413.30 | 0.72 |
|  |  | Minor_axis_length + | 0.26 | 1.30 | 0.0014 |  |  |  |  |  |
|  |  | Flatteness | -0.15 | 0.86 | 0.1094 |  |  |  |  |  |
|  |  |  |  |  |  |  |  |  |  |  |
| Local control (LC) | step_1 | clinical risk (LP_LC) | 1.00 | 2.72 | <0.0001 | -316.51 | **<0.0001** | 635.01 | 637.05 | 0.73 |
|  | step_2 | clinical risk (LP_LC) + | 0.87 | 2.38 | <0.0001 | -314.29 | **0.0353** | 632.58 | 636.67 | 0.74 |
|  |  | Minor_axis_length | 0.29 | 1.33 | 0.0249 |  |  |  |  |  |
|  | step_3 | clinical risk (LP_LC) + | 0.86 | 2.37 | <0.0001 | -313.95 | 0.4127 | 633.91 | 640.04 | 0.74 |
|  |  | Minor_axis_length + | 0.36 | 1.44 | 0.0182 |  |  |  |  |  |
|  |  | Surface_to_Volume_Ratio | 0.16 | 1.18 | 0.4048 |  |  |  |  |  |
|  |  |  |  |  |  |  |  |  |  |  |
| Regional Control (RC) | step_1 | clinical risk (LP_RC) | 0.73 | 2.08 | <0.0001 | -253.75 | **<0.0001** | 509.51 | 511.31 | 0.70 |
|  | step_2 | clinical risk (LP_RC) + | 0.67 | 1.96 | 0.0001 | -252.66 | 0.1386 | 509.31 | 512.93 | 0.71 |
|  |  | Spherical_Disproportion | 0.19 | 1.21 | 0.1178 |  |  |  |  |  |

**eTable R2.1.3** Potential clinical + geometric radiomics models

| ***Endpoint*** | ***Model*** | ***Variables*** | **MDACC (455 pts)** | **UMCG (229 pts)** | **MGH (430 pts)** |
| --- | --- | --- | --- | --- | --- |
| Overall Survival (OS) | Clinical | clinical risk (LP) | 0.72 (0.63-0.81) | 0.71 (0.62-0.81) | 0.74 (0.67-0.80) |
|  | Clinical + feature | LP + 'Minor axis length' | 0.73 (0.64-0.81) | 0.74 (0.64-0.83) | 0.74 (0.67-0.81) |
| Local control (LC) | Clinical | clinical risk (LP) | 0.73 (0.6-0.85) | 0.73 (0.5-0.96) | 0.74 (0.58-0.89) |
|  | Clinical + feature | LP + 'Minor axis length' | 0.74 (0.61-0.86) | 0.71 (0.5-0.93) | 0.73 (0.57-0.88) |
| Regional Control (RC) | Clinical | clinical risk (LP) | 0.70 (0.54-0.86) | 0.71 (0.57-0.86) | 0.73 (0.55-0.92) |
|  | Clinical + feature | LP + 'Volume density' | 0.70 (0.54-0.86) | 0.71 (0.56-0.85) | 0.74 (0.56-0.92) |
|  |  | LP + 'Spherical_Disproportion' | 0.70 (0.54-0.86) | 0.77 (0.65-0.89) | 0.76 (0.58-0.94) |

**eResults 2.2. Summary of variable selection and modelling considerations for Texture features**

For the texture radiomics features, 315 patients could be used from the training cohort, as restrictions based on metal artifacts and scanner consistency were applied. For the external validation 229 and 299 patients for external validation cohort 1 and 2, respectively.

*The univariable analyses* (***eTable R2.2.1***) in the training cohort show a significant relationship between several texture features of the primary tumor and the endpoints OS (19 out of 43 features), LC (17 out of 19 features) and RC (6 out of 43 features). For all three endpoint the most significant was ‘Second measure of information correlation’ (inf2h) from the Grey Level Co-occurrence matrix (p<0.0001). Other potential features for OS are Haralick correlation (corr_har_GLCM) and Run Emphasis (RE_GLRLM). The significance levels were lower for LC an RC, for these features.

*The forward step-wise variable selection (****eTable R2.1.2****)* was performed in the training cohort (315 MDACC patients). For OS, the ‘Sum of variance’ (sum var GLCM) added significantly (p=0.002) to both the model with the ‘clinical risk (LP) + Minor axis length’. The same feature added significantly added to the LP (p=0.02) for LC. No additional features were selected for RC.

*Bootstrapped forward selection for the Texture features* (***eFigure R2.2.1)*** in training cohort. For OS, the features that were selected in the first-step of the model building were consisted with the univariable analyses: Second measure of information correlation’ (inf2h; 346 times), Haralick correlation (corr_har_GLCM; 113 times) and the Run Emphasis (RE_GLRLM; 307 times). Additionally, the GLN_GLRLM was frequently selected (total 580 times), yet very infrequently as first variable (91 times). For RC and LC, the Second measure of information correlation’ (inf2h; LC: 436 times; RC: 418 times)) was selected primary, followed by the sum_ent_GLCM_mean with very low frequency rates (<127 times).

*The performance of potential clinical(+geometric)+texture feature models* (**eTable R2.1.3**) for the variables selected in the bootstrapped forward selection for the OS, LC and RC all did not show robust in validation. Therefore, no texture feature could reliably be selected in for the final prediction models.

**eFigure R2.2.1 Frequency plot of image variables**. Variables were selected in 1000 bootstrap sample of the training cohort


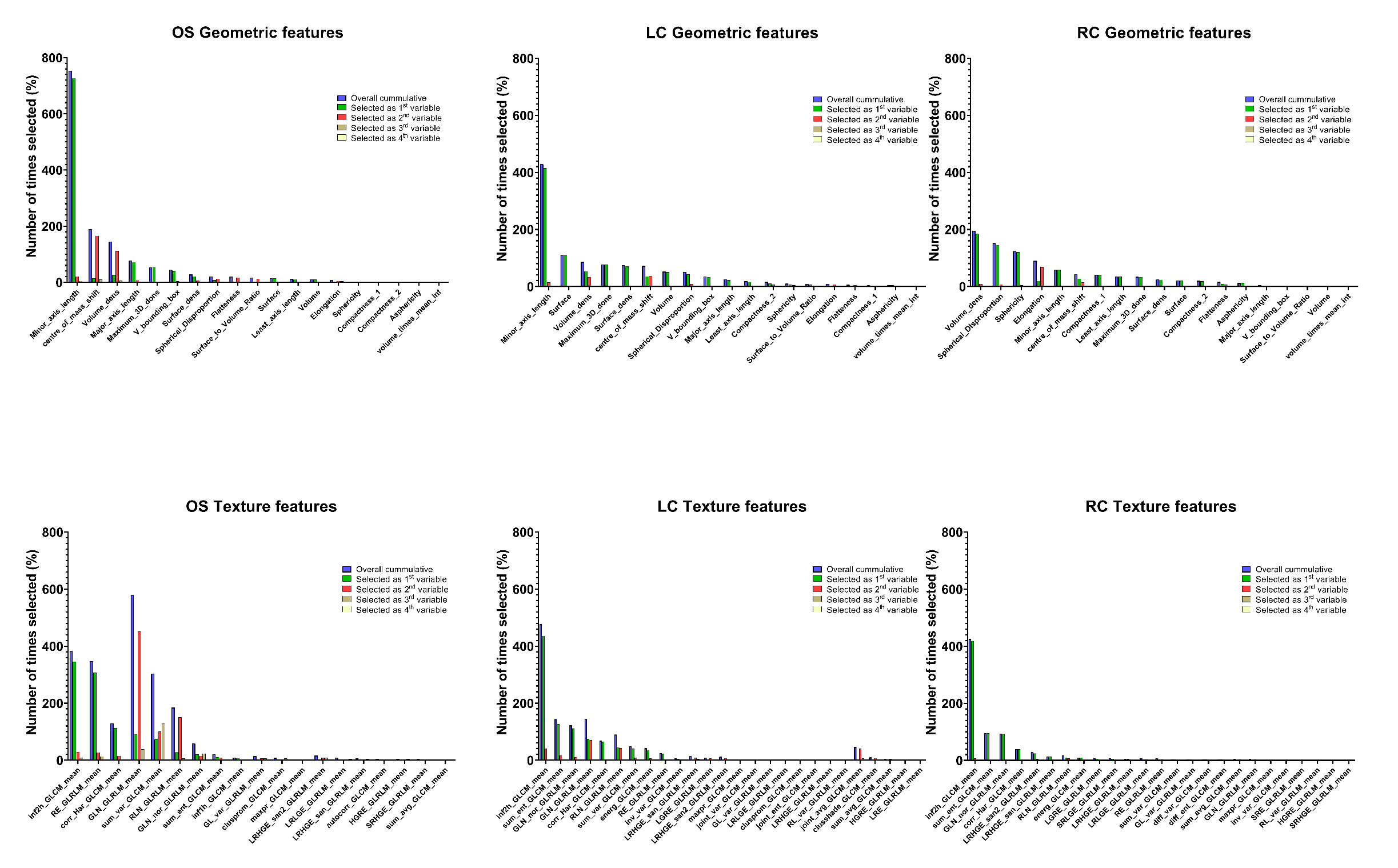


**eTable R2.2.1** Univariable analyses of texture features

| Overall survival | β | HR (95% CI) | p-value |  | Local control | β | HR (95% CI) | p-value |  | Regional Control | β | HR (95% CI) | p-value |
| --- | --- | --- | --- | --- | --- | --- | --- | --- | --- | --- | --- | --- | --- |
| **inf2h_GLCM** | 0.62 | 1.86 (1.47-2.34) | **<0.0001** |  | **inf2h_GLCM** | 0.65 | 1.91 (1.36-2.68) | **0.0001** |  | **inf2h_GLCM** | 0.52 | 1.67 (1.17-2.4) | **0.004** |
| **corr_Har_GLCM** | 0.59 | 1.8 (1.44-2.24) | **<0.0001** |  | **sum_ent_GLCM** | 0.56 | 1.75 (1.29-2.38) | **0.0004** |  | **GLN_nor_GLRLM** | -0.45 | 0.64 (0.46-0.9) | **0.009** |
| **RE_GLRLM** | 0.58 | 1.78 (1.45-2.18) | **<0.0001** |  | **GLN_nor_GLRLM** | -0.56 | 0.57 (0.42-0.78) | **0.0005** |  | **sum_ent_GLCM** | 0.44 | 1.55 (1.12-2.16) | **0.009** |
| **sum_var_GLCM** | 0.46 | 1.58 (1.33-1.88) | **<0.0001** |  | **corr_Har_GLCM** | 0.57 | 1.77 (1.29-2.43) | **0.0005** |  | **corr_Har_GLCM** | 0.41 | 1.51 (1.07-2.13) | **0.018** |
| **clustend_GLCM** | 0.46 | 1.58 (1.33-1.88) | **<0.0001** |  | **joint_ent_GLCM** | 0.46 | 1.58 (1.19-2.09) | **0.002** |  | **joint_ent_GLCM** | 0.37 | 1.44 (1.06-1.96) | **0.023** |
| **inf1h_GLCM** | -0.46 | 0.63 (0.52-0.76) | **<0.0001** |  | **sum_var_GLCM** | 0.43 | 1.53 (1.19-1.96) | **0.003** |  | **energ_GLCM** | -0.39 | 0.67 (0.46-1) | **0.040** |
| **GLN_nor_GLRLM** | -0.46 | 0.63 (0.51-0.78) | **<0.0001** |  | **clustend_GLCM** | 0.43 | 1.53 (1.19-1.96) | **0.003** |  | LRE_GLRLM | -0.47 | 0.62 (0.36-1.09) | 0.070 |
| **joint_var_GLCM** | 0.43 | 1.53 (1.29-1.82) | **<0.0001** |  | **energ_GLCM** | -0.52 | 0.59 (0.41-0.86) | **0.004** |  | sum_var_GLCM | 0.29 | 1.33 (1-1.78) | 0.076 |
| **GLN_GLRLM** | 0.37 | 1.45 (1.27-1.66) | **<0.0001** |  | **RE_GLRLM** | 0.46 | 1.59 (1.16-2.18) | **0.006** |  | clustend_GLCM | 0.29 | 1.33 (1-1.78) | 0.076 |
| **sum_ent_GLCM** | 0.45 | 1.57 (1.27-1.94) | **<0.0001** |  | **joint_var_GLCM** | 0.40 | 1.49 (1.16-1.91) | **0.006** |  | LRHGE_GLRLM | -0.43 | 0.65 (0.38-1.1) | 0.079 |
| **GL_var_GLRLM** | 0.38 | 1.47 (1.24-1.74) | **<0.0001** |  | **maxpr_GLCM** | -0.44 | 0.64 (0.46-0.91) | **0.010** |  | RE_GLRLM | 0.32 | 1.38 (0.97-1.97) | 0.079 |
| **RLN_GLRLM** | 0.34 | 1.4 (1.22-1.61) | **0.0001** |  | **inf1h_GLCM** | -0.38 | 0.68 (0.51-0.91) | **0.014** |  | RL_var_GLRLM | -0.47 | 0.63 (0.35-1.13) | 0.087 |
| **joint_ent_GLCM** | 0.35 | 1.43 (1.17-1.73) | **0.0006** |  | **RLN_GLRLM** | 0.30 | 1.35 (1.09-1.68) | **0.023** |  | joint_var_GLCM | 0.27 | 1.31 (0.98-1.75) | 0.091 |
| **clusprom_GLCM** | 0.28 | 1.32 (1.15-1.52) | **0.001** |  | **GLN_GLRLM** | 0.34 | 1.4 (1.09-1.8) | **0.025** |  | maxpr_GLCM | -0.30 | 0.74 (0.52-1.06) | 0.098 |
| **maxpr_GLCM** | -0.33 | 0.72 (0.57-0.9) | **0.004** |  | **GL_var_GLRLM** | 0.33 | 1.39 (1.07-1.81) | **0.026** |  | diff_ent_GLCM | 0.25 | 1.29 (0.97-1.71) | 0.101 |
| **energ_GLCM** | -0.33 | 0.72 (0.56-0.91) | **0.005** |  | **inv_var_GLCM** | 0.39 | 1.47 (1.01-2.15) | **0.040** |  | inf1h_GLCM | -0.27 | 0.76 (0.55-1.05) | 0.116 |
| **clusshade_GLCM** | -0.24 | 0.78 (0.67-0.91) | **0.008** |  | **diff_ent_GLCM** | 0.28 | 1.33 (1.02-1.72) | **0.048** |  | LRHGE_san_GLRLM | -0.31 | 0.73 (0.49-1.09) | 0.117 |
| **LGRE_GLRLM** | 0.27 | 1.3 (1.09-1.56) | **0.011** |  | indnc_GLCM | -0.28 | 0.76 (0.58-0.98) | 0.053 |  | GL_var_GLRLM | 0.25 | 1.28 (0.96-1.71) | 0.120 |
| **SRLGE_GLRLM** | 0.26 | 1.29 (1.08-1.54) | **0.012** |  | homom_GLCM | -0.26 | 0.77 (0.6-0.99) | 0.059 |  | inv_var_GLCM | 0.30 | 1.35 (0.91-2.01) | 0.124 |
| **diff_var_GLCM** | 0.24 | 1.28 (1.06-1.54) | **0.019** |  | diff_avg_GLCM | 0.21 | 1.24 (1-1.54) | 0.090 |  | SRE_GLRLM | 0.26 | 1.29 (0.93-1.81) | 0.134 |
| **diff_ent_GLCM** | 0.21 | 1.24 (1.03-1.49) | **0.032** |  | dissi_GLCM | 0.21 | 1.24 (1-1.54) | 0.090 |  | RLN_GLRLM | 0.20 | 1.22 (0.97-1.54) | 0.148 |
| contr_GLCM | 0.19 | 1.2 (1.02-1.41) | **0.047** |  | LRHGE_san_GLRLM | -0.30 | 0.74 (0.51-1.07) | 0.101 |  | indnc_GLCM | -0.22 | 0.8 (0.6-1.07) | 0.154 |
| diff_avg_GLCM | 0.16 | 1.17 (0.99-1.39) | 0.082 |  | SRE_GLRLM | 0.25 | 1.28 (0.94-1.76) | 0.122 |  | homom_GLCM | -0.21 | 0.81 (0.61-1.07) | 0.164 |
| dissi_GLCM | 0.16 | 1.17 (0.99-1.39) | 0.082 |  | RP_GLRLM | 0.24 | 1.28 (0.94-1.74) | 0.126 |  | LRLGE_GLRLM | -0.40 | 0.67 (0.36-1.24) | 0.171 |
| homom_GLCM | -0.16 | 0.86 (0.71-1.03) | 0.124 |  | clusprom_GLCM | 0.21 | 1.23 (0.97-1.56) | 0.132 |  | RP_GLRLM | 0.23 | 1.26 (0.91-1.75) | 0.173 |
| indnc_GLCM | -0.16 | 0.85 (0.7-1.04) | 0.133 |  | LRE_GLRLM | -0.35 | 0.7 (0.43-1.15) | 0.136 |  | LRHGE_san2_GLRLM | -0.24 | 0.78 (0.53-1.15) | 0.199 |
| homomn_GLCM | -0.14 | 0.87 (0.73-1.05) | 0.179 |  | LRHGE_GLRLM | -0.32 | 0.72 (0.46-1.15) | 0.148 |  | RLN_nor_GLRLM | 0.20 | 1.23 (0.9-1.66) | 0.206 |
| idmnc_GLCM | -0.10 | 0.9 (0.75-1.09) | 0.299 |  | SRLGE_GLRLM | 0.23 | 1.25 (0.94-1.66) | 0.158 |  | diff_avg_GLCM | 0.18 | 1.19 (0.93-1.53) | 0.213 |
| SRE_GLRLM | 0.11 | 1.12 (0.9-1.39) | 0.322 |  | diff_var_GLCM | 0.22 | 1.25 (0.94-1.65) | 0.158 |  | dissi_GLCM | 0.18 | 1.19 (0.93-1.53) | 0.213 |
| RLN_nor_GLRLM | 0.09 | 1.1 (0.9-1.35) | 0.372 |  | LRHGE_san2_GLRLM | -0.25 | 0.78 (0.55-1.11) | 0.162 |  | diff_var_GLCM | 0.18 | 1.2 (0.89-1.62) | 0.270 |
| LRLGE_GLRLM | 0.13 | 1.14 (0.84-1.53) | 0.413 |  | RLN_nor_GLRLM | 0.21 | 1.23 (0.92-1.64) | 0.168 |  | SRLGE_GLRLM | 0.17 | 1.19 (0.88-1.61) | 0.304 |
| RP_GLRLM | 0.09 | 1.09 (0.88-1.36) | 0.415 |  | LGRE_GLRLM | 0.23 | 1.26 (0.94-1.68) | 0.169 |  | SRHGE_GLRLM | 0.17 | 1.18 (0.85-1.64) | 0.321 |
| inv_var_GLCM | 0.09 | 1.1 (0.86-1.4) | 0.442 |  | contr_GLCM | 0.18 | 1.2 (0.95-1.52) | 0.181 |  | contr_GLCM | 0.15 | 1.16 (0.89-1.51) | 0.327 |
| SRHGE_GLRLM | 0.07 | 1.08 (0.86-1.34) | 0.520 |  | RL_var_GLRLM | -0.32 | 0.73 (0.44-1.21) | 0.190 |  | GLN_GLRLM | 0.15 | 1.16 (0.88-1.53) | 0.343 |
| LRHGE_san_GLRLM | -0.07 | 0.93 (0.74-1.17) | 0.547 |  | idmnc_GLCM | -0.16 | 0.85 (0.66-1.09) | 0.238 |  | idmnc_GLCM | -0.14 | 0.87 (0.66-1.14) | 0.345 |
| autocorr_GLCM | 0.07 | 1.07 (0.86-1.34) | 0.547 |  | clusshade_GLCM | -0.14 | 0.87 (0.66-1.13) | 0.337 |  | LGRE_GLRLM | 0.16 | 1.17 (0.85-1.61) | 0.372 |
| LRHGE_GLRLM | -0.05 | 0.96 (0.74-1.24) | 0.730 |  | SRHGE_GLRLM | 0.15 | 1.16 (0.85-1.59) | 0.356 |  | clusprom_GLCM | 0.13 | 1.14 (0.86-1.5) | 0.401 |
| LRE_GLRLM | -0.05 | 0.95 (0.72-1.26) | 0.733 |  | homomn_GLCM | -0.12 | 0.89 (0.67-1.17) | 0.434 |  | clusshade_GLCM | -0.12 | 0.89 (0.67-1.18) | 0.457 |
| LRHGE_san2_GLRLM | -0.04 | 0.97 (0.77-1.21) | 0.755 |  | LRLGE_GLRLM | -0.19 | 0.83 (0.49-1.4) | 0.462 |  | homomn_GLCM | -0.11 | 0.89 (0.67-1.2) | 0.489 |
| sum_avg_GLCM | 0.03 | 1.03 (0.82-1.3) | 0.776 |  | joint_avg_GLCM | -0.03 | 0.97 (0.69-1.36) | 0.842 |  | autocorr_GLCM | 0.05 | 1.05 (0.75-1.49) | 0.769 |
| joint_avg_GLCM | 0.03 | 1.03 (0.82-1.3) | 0.776 |  | sum_avg_GLCM | -0.03 | 0.97 (0.69-1.36) | 0.842 |  | HGRE_GLRLM | 0.04 | 1.05 (0.74-1.49) | 0.803 |
| RL_var_GLRLM | -0.02 | 0.98 (0.74-1.29) | 0.860 |  | autocorr_GLCM | -0.01 | 0.99 (0.71-1.38) | 0.949 |  | sum_avg_GLCM | 0.04 | 1.04 (0.73-1.48) | 0.845 |
| HGRE_GLRLM | 0.02 | 1.02 (0.81-1.28) | 0.893 |  | HGRE_GLRLM | -0.01 | 0.99 (0.71-1.39) | 0.976 |  | joint_avg_GLCM | 0.04 | 1.04 (0.73-1.48) | 0.845 |

**eTable R2.2**.**2** Forward step-wise variable selection for Texture features

| ***endpoint*** | ***modelling*** | ***variables*** | ***coef*** | ***HR*** | ***p value*** | ***LLH*** | ***ratio-test*** | ***AIC*** | ***BIC*** | ***c-index*** |
| --- | --- | --- | --- | --- | --- | --- | --- | --- | --- | --- |
| Overall Survival (OS) | step 1 | clinical risk (LP) | 1.06 | 2.88 | 0.0000 | -473.21 | 0.0000 | 948 | 951 | 0.75 |
|  | step 2 | clinical risk (LP) + | 0.95 | 2.58 | 0.0000 | -470.25 | 0.0150 | 944 | 950 | 0.75 |
|  |  | Minor axis length | 0.23 | 1.26 | 0.0089 |  |  |  |  |  |
|  | step 3 | clinical risk (LP) + | 0.85 | 2.35 | 0.0000 | -465.55 | 0.0022 | 937 | 945 | 0.75 |
|  |  | Minor axis length+ | 0.25 | 1.28 | 0.0067 |  |  |  |  |  |
|  |  | sum var GLCM mean | 0.34 | 1.41 | 0.0010 |  |  |  |  |  |
|  | step 4 | clinical risk (LP) + | 0.91 | 2.48 | 0.0000 | -463.50 | 0.0427 | 935 | 945 | 0.76 |
|  |  | Minor axis length+ | 0.17 | 1.19 | 0.0825 |  |  |  |  |  |
|  |  | sum var GLCM mean+ | 0.40 | 1.49 | 0.0001 |  |  |  |  |  |
|  |  | LRLGE GLRLM mean | 0.31 | 1.37 | 0.0325 |  |  |  |  |  |
|  | step 5 | clinical risk (LP) + | 0.88 | 2.41 | 0.0000 | -463.20 | 0.4435 | 936 | 949 | 0.76 |
|  |  | Minor axis length+ | 0.16 | 1.17 | 0.1115 |  |  |  |  |  |
|  |  | sum var GLCM mean+ | 0.34 | 1.41 | 0.0053 |  |  |  |  |  |
|  |  | LRLGE GLRLM mean+ | 0.31 | 1.36 | 0.0349 |  |  |  |  |  |
|  |  | inf2h GLCM mean | 0.10 | 1.11 | 0.4541 |  |  |  |  |  |
| Local control (LC) | step 1 | LC LP | 1.02 | 2.76 | 0.0000 | -225.06 | 0.0000 | 452 | 454 | 0.74 |
|  | step 2 | LC LP | 0.84 | 2.32 | 0.0002 | -222.30 | 0.0188 | 449 | 452 | 0.76 |
|  |  | inf2h GLCM mean | 0.41 | 1.50 | 0.0237 |  |  |  |  |  |
|  | step 3 | LC LP | 0.87 | 2.38 | 0.0001 | -221.32 | 0.1617 | 449 | 454 | 0.77 |
|  |  | inf2h GLCM mean | 0.44 | 1.55 | 0.0148 |  |  |  |  |  |
|  |  | joint avg GLCM mean | -0.24 | 0.78 | 0.1694 |  |  |  |  |  |
| Regional Control (RC) | step 1 | RC LP | 0.75 | 2.11 | 0.0000 | -197.95 | 0.0000 | 398 | 400 | 0.72 |
|  | step 2 | RC LP | 0.66 | 1.94 | 0.0004 | -196.55 | 0.0942 | 397 | 400 | 0.73 |
|  |  | inf2h GLCM mean | 0.29 | 1.34 | 0.1056 |  |  |  |  |  |

**eTable R2.2.3** Potential clinical + geometric +texture radiomics models

| ***endpoint*** |  | **MDACC (455 pts)** | **UMCG (229 pts)** | **MGH (299 pts)** |
| --- | --- | --- | --- | --- |
| Overall Survival (OS) | Clinical LP + Minor Axis Length … | 0.75 (0.65-0.85) | 0.74 (0.64-0.83) | 0.71 (0.61-0.81) |
|  | … + ‘inf2h GLCM’ | 0.76 (0.67-0.85) | 0.73 (0.63-0.82) | 0.69 (0.59-0.79) |
|  | … + ‘corr Har GLCM’ | 0.76 (0.66-0.85) | 0.75 (0.66-0.84) | 0.70 (0.59-0.8) |
|  | … + ‘sum var GLCM’ | 0.76 (0.66-0.85) | 0.73 (0.64-0.82) | 0.70 (0.6-0.8) |
| Local control (LC) | clinical LP (LC) | 0.74 (0.6-0.88) | 0.73 (0.5--0.9) | 0.55 (0.28-0.8) |
|  | … + ‘inf2h GLCM’ | 0.76 (0.63-0.88) | 0.64 (0.38-0.89) | 0.62 (0.35-0.88) |
| Regional control (RC) | clinical LP (RC) | 0.72 (0.55-0.88) | 0.71 (0.57-0.86) | 0.60 (0.31-0.89) |
|  | … + ‘inf2h GLCM’ | 0.72 (0.56-0.89) | 0.70 (0.55-0.84) | 0.66 (0.38-0.93) |
